# The relationship between autophagy and the immune system and its applications for tumor immunotherapy

**DOI:** 10.1186/s12943-019-0944-z

**Published:** 2019-01-24

**Authors:** Guan-Min Jiang, Yuan Tan, Hao Wang, Liang Peng, Hong-Tao Chen, Xiao-Jun Meng, Ling-Ling Li, Yan Liu, Wen-Fang Li, Hong Shan

**Affiliations:** 1 Department of Clinical laboratory, The Fifth Affiliated Hospital of Sun Yat-sen University, Zhuhai, Guangdong China; 2 Central Laboratory, The Fifth Affiliated Hospital of Sun Yat-sen University, Zhuhai, Guangdong China; 3 Department of Clinical Laboratory, Hunan Cancer Hospital, The Affiliated Cancer Hospital of Xiangya School of Medicine, Central South University, Changsha, Hunan China; 4 Department of Clinical Laboratory, The First Affiliated Hospital of University of Science and Technology of China, Hefei, Anhui China; 5 Department of Clinical laboratory, The Fifth Affiliated Hospital of Guangzhou Medical University, Guangzhou, Guangdong China; 6 Department of Endocrinology, The Fifth Affiliated Hospital of Sun Yat-sen University, Zhuhai, Guangdong China; 7 Key Laboratory of Biomedical Imaging of Guangdong Province, Guangdong Provincial Engineering Research Center of Molecular Imaging, The Fifth Affiliated Hospital of Sun Yat-sen University, Zhuhai, Guangdong China

**Keywords:** Autophagy, Tumor, Immune response, Immune resistance, Tumor immunotherapy

## Abstract

Autophagy is a genetically well-controlled cellular process that is tightly controlled by a set of core genes, including the family of autophagy-related genes (ATG). Autophagy is a “double-edged sword” in tumors. It can promote or suppress tumor development, which depends on the cell and tissue types and the stages of tumor. At present, tumor immunotherapy is a promising treatment strategy against tumors. Recent studies have shown that autophagy significantly controls immune responses by modulating the functions of immune cells and the production of cytokines. Conversely, some cytokines and immune cells have a great effect on the function of autophagy. Therapies aiming at autophagy to enhance the immune responses and anti-tumor effects of immunotherapy have become the prospective strategy, with enhanced antigen presentation and higher sensitivity to CTLs. However, the induction of autophagy may also benefit tumor cells escape from immune surveillance and result in intrinsic resistance against anti-tumor immunotherapy. Increasing studies have proven the optimal use of either ATG inducers or inhibitors can restrain tumor growth and progression by enhancing anti-tumor immune responses and overcoming the anti-tumor immune resistance in combination with several immunotherapeutic strategies, indicating that induction or inhibition of autophagy might show us a prospective therapeutic strategy when combined with immunotherapy. In this article, the possible mechanisms of autophagy regulating immune system, and the potential applications of autophagy in tumor immunotherapy will be discussed.

## Background

Autophagy is stimulated by cellular or environmental stresses in order to clear damaged organelles, protein aggregates, and intracellular pathogens through the formation of autophagosomes, which are subsequently targeted to lysosomal digestion. The complete macroautophagic process is generally divided into the following stages: induction, vesicle nucleation, vesicle elongation, docking and fusion, degradation, and recycling. The degraded and recycled cytoplasmic components can provide nutrients and ATP to maintain protein synthesis and other necessary metabolic functions. Thus, autophagy is considered to be an endogenous defense mechanism [1–3]**.** Most cells sustain low basal autophagy to survive under normal circumstances. In addition to cytoprotective effects, autophagy exerts a death stimulation function, known as autophagic cell death, depending on the specific conditions [4]. In the meantime, autophagy offers a therapeutic opportunity to patients with diverse diseases, such as rheumatic diseases, ischemic heart diseases, central nervous system (CNS) diseases and tumors by modulating apoptosis, inflammation, immune responses, and other intracellular processes [5–8]. Nevertheless, the defective specificity of autophagy activators or inhibitors limits their clinical applications. For instance, rapamycin acts as a autophagy activator when it was acute exposure, which leads to rapamycin 1(mTORC1) inhibition through FK506-binding protein 1A (FKBP1A), further damaging protein synthesis, mitochondrial biogenesis, and oxidative metabolism. While chronic rapamycin administration, it acts as a autophagy inhibitor promotes mTORC2 disassembly, resulting in increased lipolysis, reduced glucose uptake and activated gluconeogenesis [9, 10].

Autophagy plays a significant role in tumor promotion and suppression. However, autophagy is a “double-edged sword” in tumors, depending on the cell/tissue types and the tumor stages, which hinders the clinical application of autophagy activators or inhibitors. During the early stages of tumor development, autophagy removes damaged organelles and DNA to maintain normal cellular structure and metabolic stability to inhibit the development of tumors [5]. Epithelial-to-mesenchymal transition (EMT) is essential for tumor migration and invasion, it was reported that autophagy stimulation can hander tumor invasion and metastasis by resulting in EMT inducers degradation [11, 12]. During the advanced stages of tumors, autophagy is upregulated, and promotes tumor cell proliferation through absorb nutrients and energy driving from degraded proteins and organelles [13]. Therefore, treatment of tumors via autophagy regulation is extremely complicated.

Autophagy has been reported to modulate immune system components, mainly containing natural killer (NK) cells, macrophages, dendritic cells (DCs), and T and B lymphocytes, and has an influence on their homeostasis, survival, activation, proliferation, and differentiation, which represent innate and adaptive immune responses. Meanwhile, it also influences the release of cytokines and antibodies. Conversely, some cytokines, immunoglobulins, and immune-related cells have a great effect on the function of autophagy, such as transforming growth factor (TGF)-β, interferon (IFN)-γ, interleukin (IL)-1, IL-2, and IL-12 are autophagy inducers and IL-4, IL-10, and IL-13 are autophagy inhibitors [14]. It is well-known that surgery, radiotherapy and chemotherapy are the conventional therapies, they are efficiently used to combat tumors but have some adverse effects. Immunotherapy is developing rapidly and has become a promising treatment strategy, but further research and exploration, which is related to autophagy-modulated innate and adaptive immune system, is needed. Autophagy enhances the effect of immunotherapy by ensuring an optimal release of immunostimulatory signals via delivering antigens to the immune cells, including antigen-presenting cells and CD8 + cytotoxic T lymphocytes, and hence propels their ability to initiate an immune response, which is indispensable for the activity of several components of the immune system involved in tumor recognition and elimination. However, autophagy also inhibits immune responses to attenuate the effect of immunotherapy, which impedes the clinical development of autophagy activators or inhibitors. In brief, immunotherapy has become the major direction for future tumor treatments [15]. In this article, we discuss the mechanisms of autophagy, the relationship between autophagy and tumor development, the mechanism of the autophagy-regulating immune system, and the applications for tumor immunotherapy.

## Autophagy and its regulating mechanism

Autophagy, which is stimulated by cellular or environmental stresses, is involved in several distinct biological processes, and the regulation mechanism is complex. Briefly, when the induction signals suppress mTOR1, the macroautophagic process is triggered with the formation of Atg1/ULK complex. Then, the ULK complex binds to the phospholipids inositol triphosphate-kinase (PI3K) complex (Beclin1–hVps34–PI3K) and forms a putative mammalian pre-autophagosomal structure (PAS), possibly together with vacuole membrane protein 1 (VMP1) and Atg9, in which PI3K locally produces PI3P. Next, phagophore elongation depends on two ubiquitin-like conjugation cascades, including the Atg5-Atg12 and the microtubule-associated light chain 3 (MAP-LC3/Atg8/LC3) conjugation systems. As the phagophore elongates, it progressively engulfs a portion of the cytoplasm to form the double-membrane autophagosome by fusing on itself. Finally, the fusion of an autophagosome with a lysosome leads to the formation of an autolysosome and degradation of the loads, and the resulting macromolecules are released back into the cytosol for reuse [3, 16, 17].

**Fig. 1:**
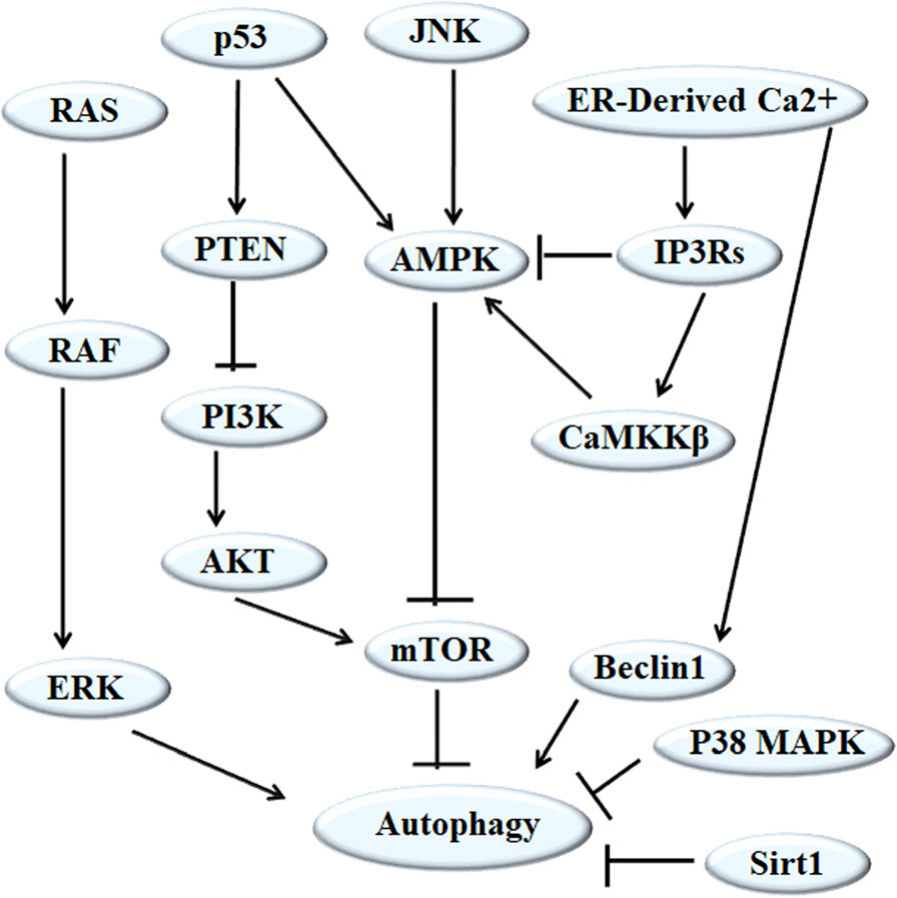
The regulating mechanism of autophagy. The signaling pathways regulating autophagy are complex. PI3K/Akt/mTOR-mediated signaling pathway inhibits autophagy, P53-activated PTEN and AMPK promotes autophagy by blocking PI3K and mTOR activation. The Ras/Raf/ERK signaling pathway induces autophagy. The JNK signaling pathway stimulates autophagy by activating AMPK. The ER-Derived Ca2+ signaling pathway enhances IP3Rs-mediated Ca2+ release, which suppresses autophagy by inhibiting AMPK activation and enhances autophagy via activating CaMKKβ and AMPK, moreover, ER-Derived Ca2+ provides Beclin1 for autophagy induction. In addition, autophagy can be blocked by sirtuin1 and P38 MAPK

In tumor cells, there are many autophagy-mediated signaling pathways. Several pathways have been reported as follows: Firstly, the activation of PI3K/Akt/mTOR-mediated signaling pathway can inhibit autophagy, which is modulated by PTEN (phosphatase and tensin homolog deleted from chromosome ten), insulin, Sirt1, 5’ AMP-activated protein kinase (AMPK), mitogen-activated protein kinase (p38-MAPK), p53, and reactive oxygen species (ROS)-associated pathways [18–20]. Secondly, the Ras/Raf/ERK signaling pathway, as one of the most commonly dysregulated pathways activated by frequently activated mutations in Ras or B-Raf oncogenes in tumors, plays a vital role in promoting autophagy [21, 22]. Thirdly, the c-Jun N-terminal kinases (JNK) signaling pathway is involved in the post-translational modification of Bcl-2 and constitutive Bcl-2 phosphorylation, which dissociates Bcl-2 from Beclin1 and stimulates autophagy [23–25]. Lastly, the intracellular calcium signaling pathway exists in the ER, mitochondria, and lysosomes. The release of Ca2+ is controlled by the inositol 1,4,5-trisphosphate receptors (IP3Rs) or ryanodine receptors (RyRs), two-pore channels 1/2 (TPC1/2), and transient receptor potential superfamily channels, such as transient receptor potential cation channel member 1 (TRPML1) and TRPM2, furthermore, ER-Derived Ca2+ released from IP3Rs can hander autophagy by inhibiting AMPK and stimulate autophagy by activating AMPK or Beclin1(Fig. 1) [26–29].

## The relationship between autophagy and tumor development

**Fig. 2:**
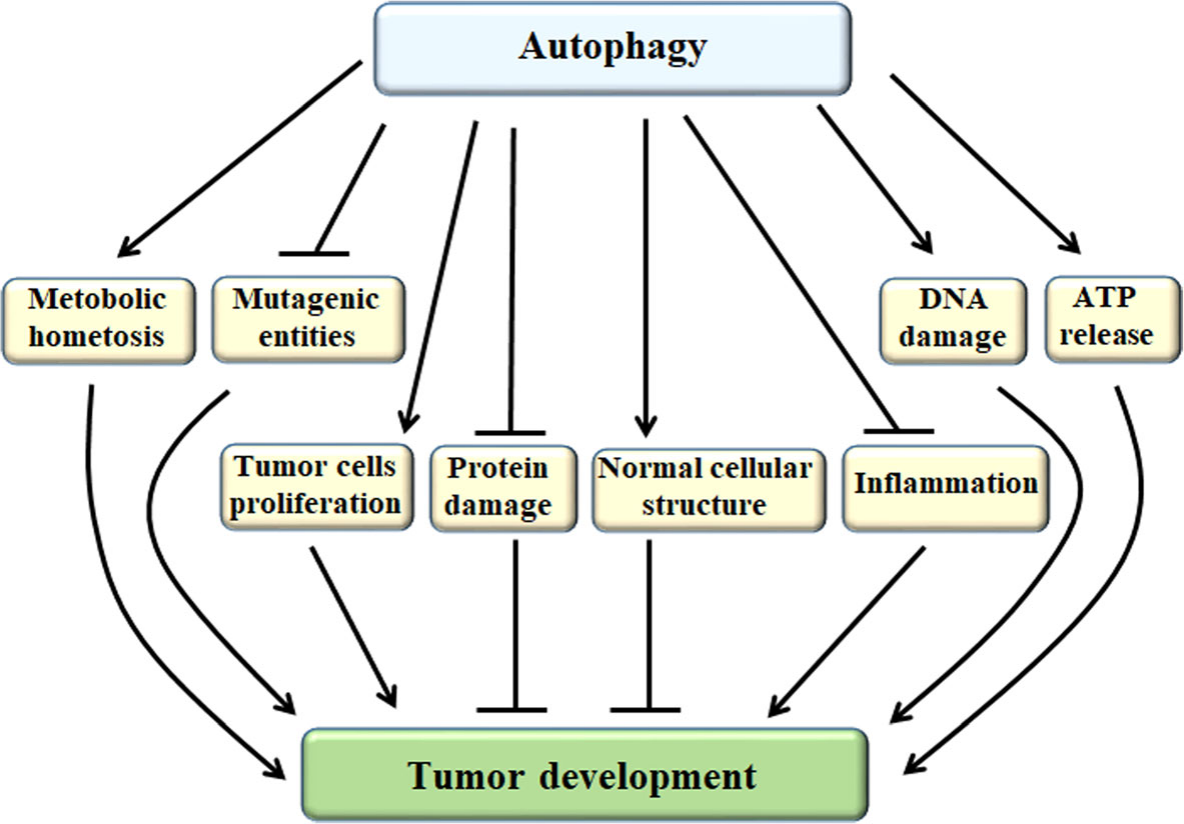
The relationship between autophagy and tumor development. Autophagy promotes tumor development by ensuring metabolic homeostasis, augmenting tumor cells proliferation, enhancing DNA damage and ATP release. On the other side, autophagy suppresses tumor development by ensuring normal cellular structure, inhibiting mutagenic entities, protein damage and inflammation

Autophagy, as a “double-edged sword” in tumors, it can promote or suppress tumor development. The regulation network of autophagy that influences tumor progression and resistance to therapy depends on the cell/tissue types and the stages of tumor, thereby inhibiting or promoting tumor formation and treatment resistance (Fig. 2). However, these complicated functions of autophagy in tumors remain to be elucidated.

In most models, tumor initiation is suppressed by autophagy via preventing the toxic accumulation of damaged proteins and organelles, particularly mitochondria. Autophagy limits oxidative stress, chronic tissue damage, and oncogenic signaling, which suppresses tumor initiation. The essential autophagy gene ATG6/BECN1 encoding the Beclin1 protein has been implicated as a tumor suppressor in breast, ovarian, and prostate cancers. Due to the important role of this gene in essential physiological and pathological pathways, it could be viewed as a putative drug target for the development of new therapies [30]. However, there is no evidence for BECN1 mutation or loss in any other tumors, suggesting that it remains uncertain whether BECN1 is a tumor suppressor in most human tumors [31]. Regardless of whether BECN1 is a tumor suppressor gene or not, another autophagy-related gene, PARK2 (Parkin), has been identified as a potential tumor suppressor that is frequently deleted in human tumors. Inactivation of PARK2 results in the accumulation of cyclin D and acceleration of cell cycle progression [32]. Defective autophagy leads to accumulation of p62, which promotes toxic accumulation of ROS and chromosomal instability, and p62 is also a signaling adaptor that regulates many oncogenic pathways, including nuclear factor erythroid 2–related factor 2 (NRF2), mTOR, and nuclear factor kappa B (NF-κB) [33]. The induction of p62 in autophagy-deficient cells alters cell function and possibly promotes tumorigenesis. Autophagy-deficient Kupffer cells promote hepatocarcinogenesis during the pre-neoplastic stage by ROS-mediated inflammation and fibrosis-promoting effects via enhancing NF-κB-IL1α/β pathways [34]. Similarly, ATG gene deletion is seen in the pancreas, as another tissue in which chronic inflammation is tumor-promoting, and stimulated benign tumor development [35]. Furthermore, autophagy plays a role in the response of death receptor agonists to block tumor cell proliferation, Fas ligand (FasL), and tumor necrosis-like apoptosis-inducing ligand (TRAIL). The canonical apoptosis receptor agonists are being tested as anti-tumor agents; however, in the same population of cells, high autophagy causes increased sensitivity to Fas-induced apoptosis but reduced sensitivity to TRAIL-induced apoptosis [36]. Apparently, this effect indicates autophagy-promoted Fas-induced apoptosis is cell type–specific. The molecular explanation is that a cell type–specific negative regulator of Fas-induced apoptosis is degraded by selective autophagy. Consequently, the pro-apoptotic effect only existed in some tumor cells [37]. Another example explains why autophagy inhibits TRAIL-induced apoptosis, in which it has been shown that autophagy can degrade activated caspase-8 to limit activation of the TRAIL apoptosis pathway [38]. Thus, autophagy has opposing effects on two very similar death stimuli (FasL and TRAIL) even in the same tumor cells.

During the process of tumors development, it has acknowledged that autophagy handered tumor invasion and metastasis by degrading EMT inducers. For instance, mTOR complexes inhibition-induced autophagy contributes to the significant decrease of SNAI family members such as SNAIL and SLUG proteins, then up-regulating cadherin and inhibiting invasion and metastasis [12]. Additionally, death effector domain-containing DNA-binding protein (DEDD) activates autophagy by directly interacting with the class III PtdIns 3-kinase complex containing PIK3C3 and BECN1, leading to the autophagy-lysosome dependent SNAI and TWIST degradation and attenuating the EMT process and the metastatic phenotype [11].

By contrast, some tumors induce autophagy and are dependent on autophagy-mediated recycling to maintain mitochondrial function and energy homeostasis to meet the metabolic demand of growth and proliferation. Moreover, tumors are more autophagy-dependent than normal tissues; thus, autophagy inhibition may be beneficial for tumor therapy [39]. p53 encoded by the *TP53* gene can regulate DNA damage response, but in stressful environments, autophagy suppresses the p53 response to promote tumor progression [40]. In this specific case, oncogenic Ras/B-Raf–triggered tumor initiation depends on autophagy to maintain healthy mitochondria and supply glutamine through lysosomal recycling. For example, oncogenic Ras-driven pancreatic tumors require autophagy in order to progress to malignant pancreatic ductal adenocarcinoma in vivo. The anti-tumor effects of inhibiting autophagy in multiple tumor types in the context of oncogenic Ras have been reported to be dependent on p53 that suppresses autophagy by inhibiting AMPK, and activating mTOR, suggesting that the loss of the tumor suppressor p53 in the context of oncogenic Ras significantly accelerates tumor cell proliferation [41, 42]. Hence, autophagy is not protective in some special conditions and stages, but is actually related to the anti-tumor effect of most of drugs. For example, it was reported that erlotinib (a standard therapy in EGFR-mutant lung cancer) induced autophagy in growth factor receptor mutated non-small cell lung cancer (NSCLC) cells, which caused drug resistance, but inhibition of autophagy by chloroquine (CQ) can enhance the pro-apoptotic effects of erlotinib [43]. Therefore, the inhibitors of autophagy may be a potential therapy strategy to overcome drug resistance.

## The relationship between autophagy and the immune system

**Fig. 3:**
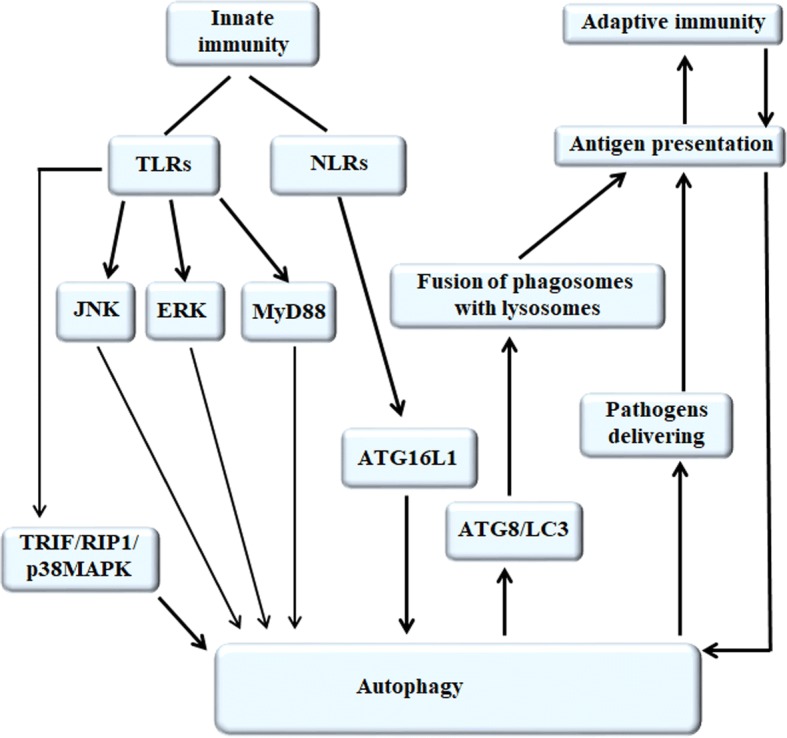
The mechanism of autophagy regulating immune system. Autophagy can be up-regulated by the activation of innate immune receptors, including TLRs and NLRs. TLRs can activate TRIF/RIP1/p38MAPK, JNK and ERK signaling pathways, or in a MyD88-dependent manner to trigger autophagy. NLRs directly induce autophagy through recruiting and interacting with ATG16L1. In adaptive immunity, autophagy can be enhanced by antigen presentation, and autophagy activation facilitates the recruitment ATG8/LC3 to phagosome membrane, the fusion of phagosomes with lysosomes and the modification of phagosomal content, contributing to increased antigen presentation and adaptive immunity

Immune system including innate immunity and adaptive immunity plays a key role in immunosurveillance of tumors. In innate immunity, autophagy works downstream of pattern recognition receptors by activation of innate immune receptors, including TLRs and NLRs, where it facilitates a number of effector responses, including NKT cell activation, cytokine production, and phagocytosis. In adaptive immunity, autophagy provides a substantial source of antigens for loading onto MHC class II molecules and it may be important in dendritic cells for cross-priming to CD8+ T cells (Fig. 3).

### Innate immunity-mediated autophagy

Innate-immunity-mediated autophagy can be upregulated by the activation of innate immune receptors, including Toll-like receptors (TLRs) and nucleotide oligomerization domain (NOD)-like receptors (NLRs) [44]. TLR2 has been reported to stimulate autophagy to enhance host innate immune responses through the activation of the JNK and ERK signaling pathways [45, 46]. TLR7 can trigger the autophagy by engaging with Atg5 and Beclin1 in a myeloid differentiation factor 88 (MyD88)-dependent manner to eliminate intracellular residues [47]. TLR4 induced autophagy via activating the TRIF (Toll-IL-1 receptor (TIR) domain-containing adapter-inducing IFN)/RIP1 (Receptor-interacting protein)/p38-MAPK signaling pathway [48]. It was reported that toll-like receptor adaptor molecule 1 (TICAM1/TRIF) was required for TLR4- and TLR3-induced autophagy stimulation by lipopolysaccharides (LPS) and polyinosinic-polycytidylic acid (poly(I: C)) respectively, which is critical for ubiquitination of TRAF6 and subsequent activation of MAPK and NF-KB signaling, and then produces unfavorable cytokines to enhance migration and invasion of malignant cells [49]. In addition to TLRs, the DNA damage-regulated autophagy modulator 1(DRAM1) mediates pathogen recognition by the TLR-MYD88-NF-κB innate immune sensing pathway to activate selective autophagy [50]. While TLRs sense microbes on the cell surface, NOD1 and NOD2, members of NLRs, recognize cytosolic pathogens by incorporating with meso-diaminopimelic acid (iE-DAP) and muramyl dipeptide (MDP), respectively. They can also activate the NF-kB and MAPK pathways to produce proinflammatory and immunosuppressive cytokines [51]. NOD1 and NOD2 directly induce autophagosome formation by recruiting and interacting with ATG16L1 [44, 52–54]. NOD1 and NOD2 in DCs could potentiate TLR-mediated invariant NKT cell activation during bacterial infection and then produce the key antibacterial cytokines, such as IFN-γ [55]. By altering the balance between pro- and anti-inflammatory cytokines, NOD1 and NOD2 modulate the risk of tumor and may bring about distinct outcomes. It has been reported that the variance of the NOD1/CARD4 (caspase recruitment domain) gene might influence lung cancer diagnosis and treatment, whereas the variance of the NOD2/CARD15 gene is not associated with lung cancer risk in the Turkish population [56]. Recently, interferon regulatory factor 8 (IRF8) has been reported to be a major regulator for autophagy maturation and innate immune responses by directly promoting autophagosome formation and lysosomal fusion [57].

### Adaptive immunity-mediated autophagy

In adaptive immunity, autophagy is essential to antigen presentation, thymus selection, lymphocyte development, and homeostasis and cytokines release, which participate in anti-tumor effects. An adaptive immune response depends on the identification of extracellular or intracellular peptide epitopes presented by major histocompatibility complex (MHCII) and MHCI molecules, which are recognized by CD4+ and CD8+ T cells, respectively [58]. T cell receptors interact with antigens presented by professional antigen-presenting cells (APCs) to initiate the humoral and cell-mediated adaptive immune responses, which promote antibody affinity maturation and cytotoxic T cells (CTLs) maintenance. In addition, autophagy provides the ATPs for anti-tumor T-cells to activate APCs. When autophagy is triggered, autophagosomes engulf intracellular pathogens and deliver degraded products to MHCII-containing compartments (MIICs) for antigen presentation to a specific CD4+ T cell. Autophagy might also facilitate the presentation of extracellular antigens to MHCII molecules by means of ATG8/LC3-associated phagocytosis (LAP). ATG8/LC3 is recruited to phagosome membranes surrounded by pathogen-associated molecular pattern (PAMP) receptors, which enhances the fusion of phagosomes with lysosomes and modifies phagosomal content [59, 60]. In *Mycobacterium tuberculosis* (Mtb)-infected dendritic cells, PE_PGRS47, as an Mtb gene, inhibits autophagy and impairs MHCII presentation of antigens. The PE_PGRS47 deletion mutant of Mtb attenuates autophagy inhibition and increases acidification and the fusion of lysosomes with phagosomes [61]. In addition, autophagy plays a role in antigen processing for MHCI cross-presentation. A study has proved that alpha-tocopheryloxyacetic acid (α-TEA) stimulates autophagy and generates autophagosome-enriched supernatant fraction (α-TAGS), as an antigen carrier which can stimulate MHCI cross-presentation to antigen-special CD8+ T cells and enhance cross-priming of CD8+ T cells [62, 63].

## The relationship between autophagy and immune cells

**Fig. 4:**
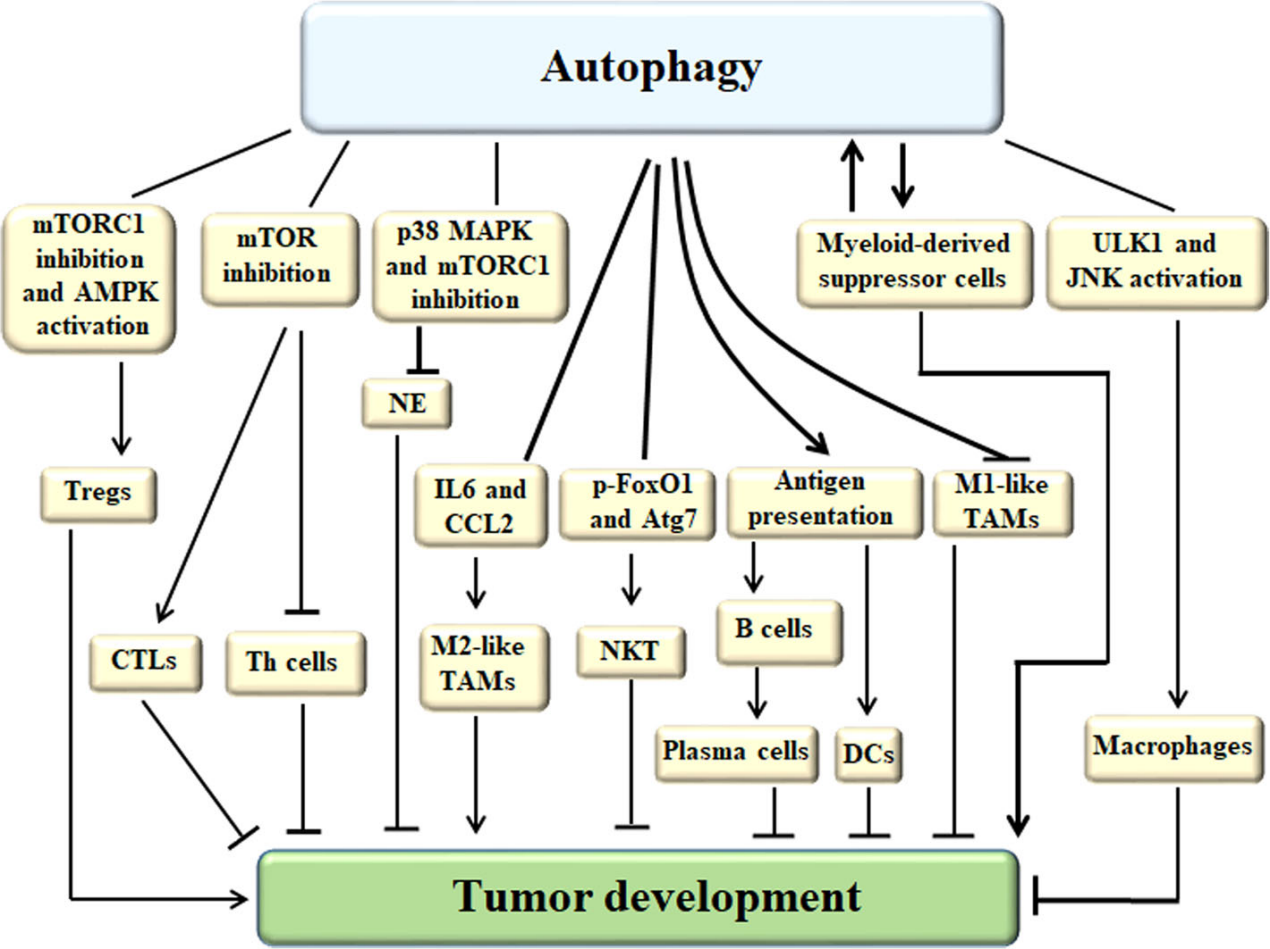
The relationship between autophagy and immune cells. Autophagy activation can promote or inhibit the development of tumor by modulating the homeostasis, activation, proliferation and differentiation of immune cells. Autophagy activated by mTOR inhibition facilitates CD8+ T cells to differentiate into CTLs, but mTOR induction promotes T cells to differentiate into Th cells. Autophagy drives DCs and B cells development, plasma cells differentiation and specific IgM and IgG production by enhancing antigen presentation. The association of cytosolic phosphorylated FoxO1 with Atg7 contributes to the autophagy induction and initiates NKT cells development and effector functions against tumor cells. mTORC1 inhibition and AMPK activation-induced autophagy plays an important role in Treg cells survival and Treg cell-mediated immune tolerance. ULK1 and JNK activation-triggered autophagy is essential for macrophage production at different stages, the inhibition of macrophages autophagy promotes M1-like TAMs polarization resulting in increased specific immune responses, however autophagy triggered by binding of IL6 and CCL2 to IL6R and CCR2, respectively, enhances macrophages polarization to the immunosuppressive M2-like TAMs. The inhibition of p38 MAPK or mTORC1 can block the development of neutrophils via inducing autophagy. In addition, autophagy can facilitate Myeloid-derived suppressor cells growth. Tregs, M2-like TAMs and Myeloid-derived suppressor cells promote tumor development, and other cells in this figure suppress tumor growth

Autophagy activation can promote or inhibit the development of tumor by modulating the homeostasis, activation, proliferation and differentiation of immune cells. Autophagy facilitates CD8+ T cells to differentiate into CTLs, promotes T cells to differentiate into Th cells. Furthermore, autophagy drives DCs and B cells development, plasma cells differentiation and specific IgM and IgG production by enhancing antigen presentation. Autophagy plays an important role in Treg cells survival and Treg cell-mediated immune tolerance, and autophagy is essential for macrophage production at different stages, the inhibition of macrophages autophagy promotes M1-like tumor-associated macrophages (TAMs) polarization resulting in increased specific immune responses, however autophagy also enhances macrophages polarization to the immunosuppressive M2-like TAMs. In addition, autophagy can facilitate Myeloid-derived suppressor cells (MDSCs) growth. While Tregs, M2-like TAMs and MDSCs promote tumor development (Fig. 4).

### T cells

Basal autophagy is reportedly required for T cells to maintain homeostasis, and defective autophagy evoked by the deletion of pro-autophagic mediators, such as Atg3, Atg5, Atg7, BECN1, and PI3K can disturb T cell survival, activation, proliferation, and differentiation [44]. Survival of naive T cells in the periphery depends on TCR interactions with stromal cells and IL-7 signaling, which appears to require Atg3-dependent autophagy in an intrinsic manner [64]. Several studies have shown that autophagy is increased in T cells after TCR stimulation, which is associated with rapidly increased calcium levels that shortly activated AMPK to promote autophagy via phosphorylation of ULK1 complex [65–67]. Atg7-deficient T cells behave like Atg5- or Atg3-deficient T cells and cannot proliferate efficiently, failing to enter into S phase after TCR stimulation. The main negative cell-cycle regulator, CDKN1B, is accumulated in naive autophagy-deficient T cells and cannot be degraded after T cell activation. However, genetic deletion of a single CDKN1B allele can restore the proliferative capacity [68]. Moreover, defective autophagy contributes to the insufficient degradation of mitochondrial components and affects the quality of mitochondria, therefore increasing ROS generation and damaging T cells [69]. Many studies have reported that the CD8+ T-cells frequency was reduced more than the CD4+ T-cells upon impaired autophagy. Inhibition of mTOR in effector CD8+ T cells can induce memory CD8+ T cells generation in lymphoid but not in mucosal tissue. According to this, we speculate that CD8+ T cells are more dependent on autophagy [70, 71]. Autophagy promotes T-cells to evolve toward invariant natural killer T (iNKT) and Treg in the thymus by regulating differentiation [72, 73]. When mTOR is induced in activated T cells, they differentiate into Th cells. Whereas mTOR activation is low and AMPK levels are high, the naive T cells preferentially differentiate into Treg cells [74]. Although all Th cells depend on mTOR activity, Th1 and Th17 cells require Rheb-dependent mTORC1 activation, whereas Th2 cells differentiation is promoted by mTORC2 activation, the genetic deletion of Rheb in T cells specifically eliminates mTORC1 activity while preserving mTORC2 activity [75]. Th17 cells differentiation is additionally regulated by the transcription factor hypoxia-inducible factor 1α (HIF-1α). The HIF1α-dependent transcriptional program is important for mediating glycolytic activity, thereby contributing to the lineage choices between TH17 and Treg cells. Lack of HIF-1α results in diminished TH17 cells development but enhances Treg cells differentiation [76]. Alternatively, autophagy may provide tumor cells with a survival advantage, protecting them against immunosurveillance by suppressing CD4+ and CD8+ T cells [77]. Th1 signature cytokines, such as IFN-γ, induce macroautophagy and accelerate not only the formation but also the maturation of autophagosomes via the JAK1/2 PI3K and p38 MAPK, but not signal transducer and activator of transcription 1 (STAT1) signaling pathways [78]. In addition, IFN-γ is a potent autophagy inducer in the Mtb-infected macrophages by enforcing phagosome-lysosome fusion [79, 80]. Conversely, Th2 cytokines, such as IL-4 and IL-13, which downregulate Th1 responses and subsequently subvert adequate protective immunity, decrease the quantity of IFN-γ and inhibit autophagy in human macrophages [81]. Surprisingly, IL-13 can induce the activation of I kappa B kinase β (IKKβ)/NFκBp65 and upregulate Beclin 1 and LC3β expression and the increase of autophagosomes in fibroblasts co-cultured with breast cancer cells [82]. IL-4 can induce autophagy in B cells, which is dependent on JAK signaling via an mTOR-independent and PI3K-dependent pathway, and promotes survival and antigen presentation of B cells [83]. Autophagy modulates the development of T lymphocytes; nevertheless, the cytokines that T cells secrete conversely promote or inhibit the progression of autophagy.

### NKT cells

Autophagy plays an essential cell-intrinsic role in maintaining the survival of a subset of innate-like cells known as iNKT cells. Phosphorylated FoxO1 and Atg7 are located in autophagosomes, suggesting that the association of cytosolic phosphorylated FoxO1 with Atg7 likely contributes to the autophagy induction of iNKT cells indicated by MAP1LC3β/LC3β lipidation and sequestosome 1(SQSTM1/p62) degradation, initiating NKT cells development and effector functions against viral infection [84]. Autophagy-deficient iNKT cells accumulate mitochondria and oxygen radicals and subsequently die of apoptosis. Furthermore, deletion in autophagy genes not only interferes with the mature stages of iNKT cells and decreases the proliferation of NKT cells, but also prevents transition to a quiescent state after population expansion [85]. Once activated by strong antigens, the majority of iNKT cells rapidly release large amounts of both Th1 and Th2 cytokines; however, autophagy-deficient iNKT cells exhibit decreased IL-4 and IFN-γ levels [86]. NKT cells exert anti-tumor effects by direct killing of tumor cells, induction of cell apoptosis, secretion of IFN-γ, and inhibition of tumor metabolism. Recent studies have demonstrated that NKT cells can induce autophagy in tumor cells because lymphocyte-to-tumor cell contact strongly enhances lymphocyte-mediated autophagy by releasing diffusible factors [87].

### Treg cells

Treg cells (FOXP3(+) regulatory T cells) require autophagy to suppress anti-tumor immune responses. Autophagy is essential for Treg cells survival, lineage stability, and Treg cells-mediated immune modulation. Treg cells-specific deletion of Atg7 or Atg5, two essential genes in autophagy, leads to loss of Treg cells, further tumor resistance, and inflammatory disorders. Research has found Treg cells have higher autophagy activity than naive CD4 + T cells. Mechanistically, autophagy-deficient Treg cells have increased apoptosis and lost characteristic expression of Foxp3 by upregulating mTORC1. The reason is that autophagy plays an important role in restricting mTORC1 activation. mTORC1 is a crucial regulator in Treg cells, and either diminished or excessive mTORC1 disrupts Treg cells suppressive functions [88, 89]. In a model of KRas-driven lung carcinogenesis, autophagy deficiency is induced by knockdown of Atg5 or Atg7, which induces the inopportune recruitment of Treg cells via ENTPD1 (CD39, an ecto-enzyme that is exposed on the cell surface)-mediated conversion of immunostimulatory ATP into immunosuppressive ADP (adenosine diphosphate 1) and AMP (adenosine monophosphate) to suppress the specific anti-tumor responses [90]. Therefore, we can design the strategies for tumor treatment using the inhibitors of ENTPD1 or the inducer of autophagy to decrease the recruitment of Treg cells. As we known, Foxp3+ T cells express variable amounts of Treg cells-related cytokines, including IL-10, TGF-β and Foxp3. IL-10 inhibits autophagy via PI3K/Akt/mTORC1 and JAK-STAT3 activation. However, TGF-β triggers autophagy via activating the Smad and JNK signaling pathways [91]. Foxp3 is a prerequisite for retroviral protein tax induction of T cell transformation, and autophagy molecules are required for maintaining tax transformation of Foxp3+ T cells, silencing key autophagy molecules, including Beclin1, Atg5, and PI3KCIII, which will result in impairing peripheral maintenance and the function of CD4 + FoxP3+ regulatory T cells [92, 93].

### B cells

Autophagy plays a role in B cell development and survival. It is dispensable for the transition between pro- and pre-B cell stage and B cell activation in response to BCR stimulation. Moreover, basal levels of autophagy are necessary to maintain a normal number of peripheral B cells and their survival after ligand lipopolysaccharide (LPS) stimulation that drives plasmablast differentiation and specific IgM and IgG production [94]. Autophagy-related genes, especially, Atg5 is essential for B cell development [44]. Tumor-derived autophagosomes (termed “DRibbles”) induce B cell activation, resulting in antibody production and cytokine secretion. The autophagy-deficient B cells lack the ability to produce antibodies and cytokines. Unexpectedly, it has been reported that unfractionated splenocytes produce a higher level of antibodies and cytokines than purified B cells, DRibbles stimulation upregulates CD40L expression on macrophages, resulting in increased level of CD40 on B cells. The accessory role of macrophages in DRibbles-activated B cells is critically dependent on the CD40/CD40L interaction. Moreover, macrophages are able to enhance the antigen presentation function of B cells for specific T cell stimulation [95]. B cell intrinsic autophagy is required for the function and/or survival of alloreactive B memory cells. During autophagy induction, LC3 molecules link to nascent autophagosome membranes in memory B cells, contributing to LC3 abundance and increasing the percentage of autophagosome-containing cells in memory B cells compared with naive B cells, which can explain why the memory B cells have a longer lifespan, and the lack of autophagy in B cells does not affect primary alloantibody responses, but affects secondary alloantibody production [96]. Therefore, pharmacological inhibitors of autophagy impair antibody recall response.

### DCs

Foxp3+ Treg cells potently impair the autophagic machinery in DCs in a CTLA4-dependent manner. Mechanistically, CTLA-4 binding to CTLA-4 antibody promotes the activation of the PI3K/Akt/mTOR axis that results in FoxO1 nuclear exclusion in DCs, leading to decreased transcription of the LC3β and the formation of autophagosomes [97]. Deficient autophagy in DCs impaired cytokines secretion, such as absence of Atg5 but not Atg7 deficiency in DCs, impaired IL-2 and IFN-γ production by CD4+ T cells in an IL-1β independent manner. However, Atg5-deficient DCs exhibited unimpaired production of IL-12, IL-6, and TNF-α [98]. Autophagy is important for the presentation of cytosolic antigens on MHCII and efficient cross-presentation of soluble antigen. A study revealed that Atg5 deficiency in DCs impaired antigen presentation through the MHCII pathway. The reason for this might be that the fusion of lysosomes with phagosomes is delayed, suggesting that Atg5 is also important for autophagosome formation and is required for DCs to trigger CD4+ T cells responses via MHCII antigen presentation, especially triggering protective antiviral Th1 cell responses [99, 100].

### Macrophages

Autophagy is essential for controlling macrophage production at different stages, including hematopoietic stem cell maintenance, monocyte/macrophage migration, monocyte differentiation into macrophages and polarization, in most solid tumors. TAMs density is significantly higher than the surrounding normal tissues. Generally, TAMs first originate from monocytes that are recruited into tumors by chemoattractants, including chemokines and cytokines released from both tumor cells and stromal cells. Among these chemoattractants, chemokine [C–C motif] ligand 2 (CCL2) exerts a prominent action in recruiting monocytes and is able to protect monocytes against apoptosis in the tumor microenvironment by upregulating anti-apoptotic proteins and inhibiting CASP8/caspase-8 cleavage, and it also induces hyper-activation of autophagy in TAMs [101]. When monocytes are stimulated to differentiate into macrophages, colony-stimulating factor 1 (CSF1) increases the expression and phosphorylation status of ULK1, thus contributing to increased induction of autophagy [102]. CSF2 is also able to promote monocyte survival and differentiation into macrophages. The differentiation signal helps Beclin1 release from Bcl-2 by activating JNK and blocks Atg5 cleavage, thus stimulating autophagy, whereas blockade of autophagy has an inhibitory effect on CSF2-induced monocyte differentiation into macrophages [103]. Autophagy also plays a key role in macrophage polarization. The inhibition of macrophage autophagy promotes M1 polarization, resulting in increased pro-inflammatory cytokine secretion. Then, M1 macrophages stimulate a Th1 response against intracellular microorganisms and tumor cells by activating immune responses, whereas the induction of autophagy promotes M2 polarization. In the tumor microenvironment, autophagy is triggered by binding of IL-6 and CCL2 to interleukin 6 receptor (IL-6R) and CCR2, respectively, which is essential for macrophage polarizaton to the M2 phenotype, resulting in increased anti-inflammatory cytokine secretion, which promotes the fading of inflammation as well as tissue repair and remodeling, but M2 macrophages are immunosuppressive cells [101, 104–106]. Targeting the autophagy which regulates macrophage polarization toward the M1 phenotype should be a promising anti-tumor strategy.

### Neutrophils

Autophagy has a negative effect on the development of neutrophils. Deficient autophagy indicates an increased proliferation rate in the neutrophil precursor cells of the bone marrow and accelerates the process of neutrophil differentiation, resulting in the accumulation of mature neutrophils in the bone marrow, blood, spleen, and lymph nodes. Pharmacological inhibition of p38 MAPK or mTORC1 can induce autophagy in neutrophilic precursor cells and block their differentiation [107]. However, autophagy is required for neutrophil-mediated inflammation. Autophagy deficiency in neutrophils leads to reduced nicotinamide adenine dinucleotide phosphate (NADPH) oxidase-mediated ROS production and further contribute to reduced degranulation [108]. Recent studies have revealed that autophagic activity is also required for the release of neutrophil extracellular traps (NETs), representing a distinct form of active neutrophil death, namely NETosis. NET formation requires both autophagic activity and ROS production. Inhibition of the mTOR pathway accelerates the rate of NET release and stimulates ROS production following neutrophil stimulation with the bacteria-derived peptide formyl-Met-Leu-Phe (fMLP) [109]. Tumor-derived neutrophils exhibit much higher levels of LC3-II and have more autophagosomes than their counterparts from blood. There is evidence that enhancement of neutrophil autophagy in hepatocellular carcinoma (HCC) is correlated with the release of matrix metalloproteinase-9 (MMP9) and oncostatin M (OSM), but is unrelated to the deactivation of mTOR signaling, which could contribute to the advanced migration of tumor cells. According to these studies, abolishing autophagy initiation by inhibiting the activation of Erk1/2, p38, and NF-κB signals in tumor-derived neutrophils could rapidly restore the spontaneous apoptosis of cells, which provides a novel strategy for anti-tumor therapy [110]. Neutrophils have the highest expression of IgA Fc receptor FcRI (CD89) of all cell types. Co-culturing of tumor cells, neutrophils, and IgA results in significant changes in the cell morphology of tumor cells, which is associated with high LC3-II expression in autophagosomes, but cell apoptosis remains constant. These phenomena suggest that autophagy participates in the process of activated neutrophil combat against tumor cells [111].

### MDSCs

MDSCs are immune-suppressive cells and their accumulation and suppressive activity are driven by inflammation. It was reported that high mobility group box 1(HMGB1) can promote the survival of MDSCs by inducing autophagy [112]. In addition, glycolytic metabolism has an essential impact on MDSCs. Glycolysis prevents the AMPK-ULK1 signaling activation and autophagy formation to enhance autophagy-mediated partial liver-enriched activator protein (LAP) expression, which in turn promotes granulocyte colony-stimulating factor (G-CSF) and granulocyte macrophage colony-stimulating factor (GM-CSF) expression and supports MDSCs development in tumors [113]. Furthermore, MDSCs are identified to induce AMPK phosphorylation, stimulate autophagy and increase the anti-apoptotic factors MCL-1 and BCL-2, which promotes Multiple Myeloma (MM) progression [114].

## The relationship between autophagy and cytokines

**Fig. 5:**
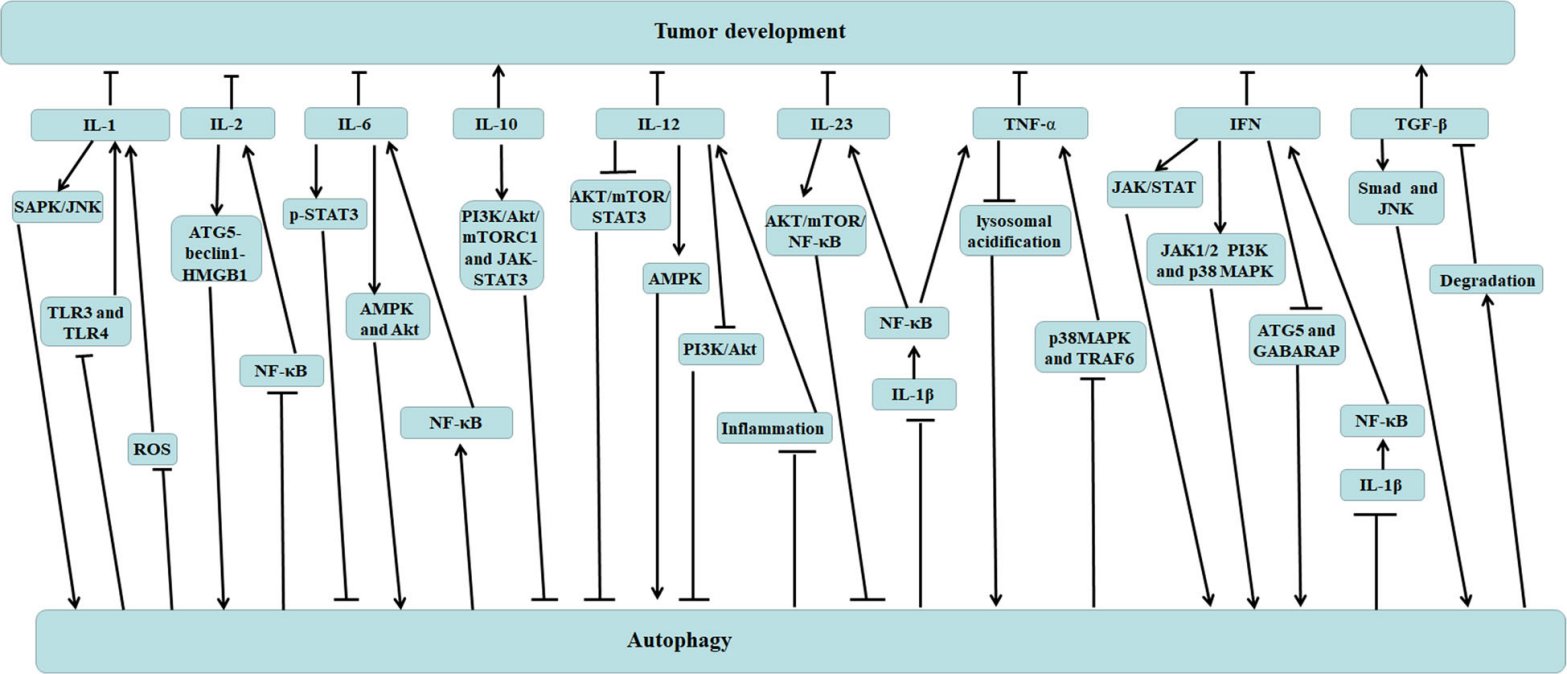
The relationship between autophagy and cytokines. Autophagy activation can promote or inhibit the secretion of cytokines to control tumor development. IL-1 can induce autophagy by SAPK/JNK signaling pathway, and autophagy represents a negative impact on IL-1 production by inhibiting the interaction between TLR4, TLR3, LPS and ROS accumulation, resulting in NF-κB signaling pathway inhibition. IL-2 boosts autophagy induction by promoting ATG5-beclin1-HMGB1 complex formation, however, autophay suppresses NF-κB-mediated IL-2 production. IL-6 exerts anti-autophagic effects by activating p-STAT3 and reducing the protein levels of LC3-II and Beclin 1, in addition, IL-6 promotes autophagy AMPK activation and mTORC1 inhibition, and Akt activation. Mutually, autophagy promotes the release of IL-6 by activating NF-κB pathway. IL-10 activates the JAK/STAT3 and PI3K/Akt/mTORC1 pathways, resulting in autophagy inhibition. IL-12 induces autophagy through suppressing the AKT/mTOR/STAT3 and PI3K/Akt pathways and activating AMPK pathway, and autophagy decreases IL-12 release by inhibiting inflammation. IL-23 contributes to autophagy inhibition and ROS accumulation by triggering AKT/mTOR/NF-κB pathway, and autophagy decreases the production of IL-23, TNFα and IFN by inhibiting IL-1β-mediated NF-κB signaling pathway. TNF represses autophagy by decreasing lysosomal acidification, and autophagy inhibits TNF-α expression through blocking p38MAPK phosphorylation and TRAF6 expression. IFNs induce autophagy by activating JAK/STAT and JAK1/2, PI3K and p38MAPK pathways, and suppress autophagy by decreasing autophagosome formation and the expression of autophagy-related genes ATG5 and GABARAP. TGF-β has been demonstrated to activate autophagy by Smads and JNK signal pathways, but autophagy decreases mature TGF-β protein levels as a result of increased degradation. In this figure, IL-10 and TGF-β enhance tumor development, other cytokines suppress tumor development

Autophagy is closely intertwined with inflammatory and immune responses, and cytokines may help mediate this interaction. Autophagy has been shown to regulate, and be regulated by, a wide range of cytokines, and autophagy activation can promote or inhibit the secretion of cytokines to control tumor development (Fig. 5).

### Il-1

The two main pro-inflammatory cytokines are IL-1α and IL-1β. IL-1 can inhibit signaling pathways, such as cyclooxygenase (COX-1), phosphorylated inhibitor of kB (IkB), and stress-activated protein kinase (SAPK)/ JNK, thereby inducing autophagy and promoting tumor development, growth, and metastasis. Inhibition of IL-1 expression in tumor cells can induce upregulation of p21 and p53, leading to suppression of tumor growth [115]. Intracellular IL-1β is targeted by autophagosomes. Autophagy has dual effects on inflammasome activation and IL-1β secretion. However, the negative effect is predominant under stable conditions, whereas only the negative role of autophagy in IL-1α activation has been reported [116, 117]. It was reported that Atg5-deficient macrophages secrete reduced amounts of IL-1β upon autophagy stimulation, restricted T cells activation, and cytokine production [98]. The production and release of mature IL-1β requires two distinct signals. The first signal is the interaction between TLR4 and TLR3 and LPS, which activates NF-κB-dependent transcription of the IL-1β gene and secreting minimal amounts of mature IL-1β. The second signal is the potassium-proton ionophore, ATP, which induces greatly increased levels of extracellular IL-1β that is dependent on caspase-1 [118]. During the pre-neoplastic stage of hepatocarcinogenesis, autophagy-deficient macrophages increase IL-1α/β production by enhancing the ROS-NF-kβ pathway [34]. Both IL-1β and IL-1α can induce autophagy; however, autophagy might limit their secretion, indicating that autophagy represents a negative feedback mechanism for controlling IL-1β and IL-1α secretion [119].

### IFN

IFN includes type I, type II, and Type III IFN. Type I IFN includes IFN-α and IFN-β, which are secreted by mononuclear phagocytes and fibroblasts, respectively. It has been reported that type I IFN induced autophagy by activating JAK/STAT via phosphorylation of STAT1 and STAT2, which was activated by tyrosine kinase (TYK) 2 and JAK1, and then implicated the involvement of MAPK signaling and the PI3K/AKT/mTOR signaling axis, accompanied by the inactivation of mTORC1 signaling and activation of mTORC2/AKt signaling via FOXO3 regulation, which is a direct transcription regulator of autophagy genes [78, 120, 121]. However, the effect of IFN-α depends on the specific targeted cell type. IFN-α can inhibit autophagy when combined with lymphocyte co-culture, although it contributes to greater MHC-1 increases [87]. IFN-γ is the Type II IFN, and it is produced by activated CD4+ and CD8+ T cells as well as NK cells. It can induce autophagy in various cell types, including epithelial cells, immune cells, and tumor cells. On the one hand, IFN-γ accelerates not only the formation but also the maturation of autophagosomes via JAK1/2, PI3K signaling cascades and the p38 MAPK signaling pathway, which is a STAT1-independent pathway. On the other hand, IFN-γ rapidly and consistently leads to the upregulation of MHCI on the surface and induces autophagy [78, 122]. Increased autophagy also enhances the process of viral/bacterial digestion. IL-27 inhibits IFN-γ-induced autophagy by concomitant induction of the JAK/PI3K/Akt/mTOR cascade, in addition, Th2 cytokines IL-4 and IL-13 can also inhibit IFN-γ-induced autophagy [14, 81, 123]. Conversely, IFN-γ is considered as a regulator to Th2 responses, and deletion of IFN-γ gene strongly triggers Th2 cytokines secretion in inflammatory diseases [124]. Type III IFNs (IFN-λs) share some common characteristics and therapeutic benefits with type I IFNs, but the effects on cellular autophagy are different from type I IFNs. IFN-λ1, the main type III IFNs produced by hepatocytes during acute HCV infection, can suppress HCV-induced autophagy indicated by decreased conversion of LC3β-I to LC3β-II amounts, decreased autophagosome formation, and decreased expression of autophagy-related genes ATG5 and GABARAP [125].

### Il-6

In vitro, IL-6 exerts anti-autophagic effects by activating the phosphorylation of STAT3 at Tyr705 and reduces the protein levels of LC3-II and Beclin 1. Treatment with a STAT3 inhibitor can reverse the inhibitory effect of IL-6 on autophagy, as activated STAT3 binds to the promoter of Bcl-2 and leads to its overexpression, which in turn reacts with Beclin1 to inhibit the formation of the Beclin1-VPS34-Atg14-p150 complex to decrease autophagy [126]. For instance, IL-6 inhibits the formation of IFN-γ and starvation-induced autophagosomes in virulent M. tuberculosis H37Rv-infected macrophages by indicating the decreased LC-II and Beclin1. IL-6 inhibits autophagy via promoting phosphorylation and expression of mTOR substrate, then inhibits phosphorylation of both p38 MAPK and JNK/SAPK induced by IFN-γ [127]. In vivo, IL-6 trans-signaling promotes autophagy by stimulating a robust increase in lysosomes but not autophagosomes. The process is dependent on IL-6R expression. Autophagy is accelerated when IL-6 is complexed with soluble IL-6R and thereafter locates to gp130 on cellular membranes. The stimulation of autophagy by IL-6 is regulated via multiple complementary mechanisms, including two main signals: one is activating AMPK, which inhibits mTORC1; the other is activating Akt, then phosphorylating STAT3 at S727 and activating Atg4c and mTORC2, ultimately leading to autophagy-related enzyme production to induce autophagy [128]. In lung cancer patients, cachexia is prevalent. There is a positive correlation between IL-6 trans-signaling-induced autophagy in the tumor and weight loss [129]. Mutually, autophagy promotes the release of IL-6 by HBV X protein (HBx)-induced autophagy and activates the NF-κB pathway. Autophagy inhibition abrogates NF-κB activation and IL-6 production [130].

### Il-2

The binding between ATG5, HMGB1 and Beclin1 is essential for IL-2-induced autophagy. Autophagy inhibitors or knockdown of ATG5 and Beclin1 can block IL-2-induced autophagy and switch IL-2-induced proliferation to apoptosis [131]. High-dose IL-2 (HDIL-2) alone increases serum levels of IFN-γ, IL-6, and IL-18 and translocates HMGB1 from the nucleus to the cytosol in hepatocytes. Then, the interaction between HMGB1 and Beclin1 boosts autophagy. However, the effects could be inhibited by combining with autophagy inhibitor Chloroquine(CQ), which inhibits autophagy by blocking acidification of the lysosome, preventing fusion with the autophagosome. In tumor cells, CQ increases autophagic vacuoles and LC3-II levels, inhibits oxidative phosphorylation and ATP production, and promotes apoptosis, suggesting that the combination of IL-2 with CQ promotes anti-tumor effects, increases long-term survival, decreases toxicity associated with vascular leakage, and enhances immune cell proliferation and infiltration in the liver and spleen [132, 133].

### Il-12

IL-12 is important for immune responses and anti-tumor activity. It can induce autophagy through AKT/mTOR/STAT3 signaling pathways via decreasing the expressions of p-AKT, p-mTOR, and p-STAT3 and inhibit hepatoma cell growth. However, the induction of autophagy attenuated the growth-inhibitory effect of IL-12 on hepatoma cells, indicating that restraining autophagy by inhibitors or silencing Beclin1 could enhance IL-12-mediated anti-tumor effects. Furthermore, in human breast cancer cells, IL-12 induces autophagy through inhibiting AMPK and activating the PI3K/Akt signaling pathway [134]. IL-12 is produced by activated inflammatory cells, therefore, autophagy decreases the release of IL-12 by inhibiting inflammation.

### Il-10

IL-10 inhibits autophagy by activating the JAK/STAT3 and PI3K/Akt/mTORC1 pathways [135]. The PI3K pathway promotes phosphorylation of p70S6K through the activation of Akt and mTORC1 [136]. A study has indicated that IL-10 inhibits angiotensin II-induced pathological autophagy by activating PI3K/Akt/mTORC1 signaling and promoting Bcl2-Beclin1 interaction that could attenuate the anti-apoptosis effect; however, pharmacological or molecular inhibitors of Akt and mTORC1 signaling can weaken IL-10-inhibited Ang II-induced autophagy [137]. Furthermore, IL-4 and IL-13 signaling also activate PI3K signaling to activate mTORC1 in macrophage cells, and Th2 cytokines IL-4, IL-13 and IL-10 exert autophagy inhibition in most environments [124]. During monocytes–DCs differentiation and DCs survival, cytoprotective autophagy responses are essential for counteracting IL-10-triggered apoptosis, but IL-10 can strongly inhibit starvation-induced autophagy and decrease Bcl-2 levels, which indicates increased levels of Beclin-1, LC3, and mature autophagosomes and results in restricting DCs growth. IL-10 not only kills nascent antigen-presenting DCs but also specifically skews the growth of DCs toward non-antigen-presenting monocytes–macrophages, and the autophagy inhibitor 3-methyladenine(3-MA) restricted DCs differentiation by prompting apoptosis [138]**.** Celastrol has been reported to ameliorate colitis in IL-10 deficient mice, which has clearly confirmed that celastrol up-regulated the autophagy of colon tissue in IL-10−/− mice by inhibiting the PI3K/Akt/mTOR signaling pathway. This could be a therapeutic target for Crohn’s disease [139]. Autophagy is likely to have dual effects on IL-10 production, but the mechanisms require further exploration [140, 141].

### TNF-α

TNF-α inhibited autophagy via disrupting the autophagic flux by decreasing lysosomal acidification, but it was reported that the increased amount of LC3-II protein level, associated with the increased of P62 protein level, without altering P62 mRNA levels. TNF-α is shown to induce apoptosis or necrosis in various types of cells, results have revealed a novel effect of TNF-α on DA neuron dysfunction and subsequent neuroinflammation-induced neuron degeneration in Parkinson’s disease [142]. Inhibition of autophagy and promotion of lysosomal proteolysis accelerate TNF-α-induced death by increasing oxidative stress and toxicity because LPS-induced TNF-α mRNA expression is further significantly enhanced by pretreatment with Bafilomycin A1, implying autophagy plays an inhibitory role in the expression of TNF-α. Autophagy causes the inhibition of p38MAPK phosphorylation and TRAF6 expression, which are required for the expression of TNF-α [143, 144].

### TGF-β

In immune evasion, TGF-β exerts suppressive effects directly on effector cells (including cytotoxic cells) and indirectly promotes the differentiation of regulatory T cells. In the tumor immunosuppressive microenvironment, TGF-β can inhibit NK cells to diminish targeted cell lysis and IFN-γ production. However, NK cells in the tumor microenvironment may restore their activity by TGF-β blockade with anti-TGF-β antibodies and small molecule inhibitors of TGF-β signaling [145]. Furthermore, inhibition of autophagy increases mature TGF-β protein levels without inducing TGF-β mRNA expression, indicating that the increase of mature TGF-β protein levels is a result of decreased degradation rather than increased synthesis [146]. Blocking TGF-β in the co-culture diminishes targeted cells autophagy in a dose-dependent manner, indicating that TGF-β might be responsible for autophagy induction [87, 145]. Recently, TGF-β has been demonstrated to activate autophagy in certain HCC and breast cancer cells, which undergo cell cycle arrest and apoptosis in response to TGF-β. In those malignant cells, TGF-β stimulates the expression of mRNA transcripts of several autophagy-related genes, such as Beclin1, Atg5, Atg7, and death-associated protein kinase (Dapk), and induces accumulation of autophagosomes and activation of autophagic flux. Up regulation of these genes is regulated by the Smad and non-Smad signal transduction pathways, including ERK, JNK, p38MAPK, and PI3K. Meanwhile, autophagy potentiates the induction of the proapoptotic Bcl-2 family protein Bim and contributes to Bim-mediated apoptosis in hematopoietic cells. TGF-β could also induce directly proapoptotic genes, Bim and Bmf, in a p38 MAPK and Smads-dependent manner, indicating a functional link between autophagy and apoptosis [147, 148].

### Il-23

Inhibitor of autophagy, such as 3-MA as a PI3K inhibitor, can block autophagic degradation of proteins and enhance LPS-induced secretion of IL-23. Moreover, knockdown of either Beclin1 or Atg7 enhances secretion of IL-23 at the transcriptional level. In autophagy-deficient cells, IL-23 secretion is directly regulated by IL-1 signaling and is dependent on the generation of ROS because ROS promoted activation of the inflammasomes, and the secretion of IL-1 and IL-1R1 is known to activate the NF-κB pathway, then stimulate the production of IL-23 as well as TNF-α [119]. The expression levels of IL-23 and IL-23R are enlarged in Hashimoto’s thyroiditis disease, which contributes to autophagy suppression and ROS accumulation by inducing AKT/mTOR/NF-κB signaling activation [149].

## The relationship between autophagy and tumor immune tolerance

Immunotherapeutic strategies aimed at boosting antitumor immunity are promising candidates for the treatment of tumors. However, the clinical outcomes of these immunotherapeutic strategies have been less effective than anticipated. Immune tolerance to these tumors is still a major impediment in cancer immunotherapy. As immunologic tolerance molecules, Indoleamine 2,3 dioxygenase(IDO), CTLA-4 and PD-1 can regulate tumor immune tolerance through autophagy pathways. Therefore, understanding the relationship between autophagy and tumor immune tolerance is important for developing tumor immunotherapy strategies.

### IDO

IDO is produced by tumor cells, tumor-associated MDSCs and TAMs. It is thought to potently suppress cytotoxic T cell responses and inflammatory dendritic cell maturation, magnify tolerogenic APCs, and promote the generation of Tregs from naive CD4 + T cells, thereby inhibiting effective anti-tumor immunity, driving immunologic tolerance, and promoting the development of tumor. Autophagy can inhibit the inflammation-mediated expression IDO production by suppressing inflammation [150–152]. General control nonderepressible 2 (GCN2) can be triggered by IDO-mediated tryptophan (Trp) deficiency, which is recognized as an important effector of the IDO pathway, resulting in auto-phosphorylation and activation of kinase activity that inhibits the translation initiation factor 2α (eIF2α), blocking protein synthesis and arresting cell growth. GCN2 is essential for inflammatory carcinogenesis [153]. When autophagy is induced by IDO or GCN2, it protect organisms from fatal inflammation disease; therefore, IDO1-GCN2-autophagy signals may be a common circuit induced in human inflammatory disease, which could be potentially targeted for therapeutic benefit [154]. Furthermore, IDO inhibits a tryptophan sufficiency signal, resulting in the inhibition of mTOR, leading to autophagy via LC3 production, and translational blockade via s6K inactivation. Tryptophan and the experimental agent 1-methyl-D-tryptophan (D-1MT, as a mimetic of Trp) functionally reverse the effects of IDO on mTOR and autophagy in the sufficiency pathway, but do not affect GCN2 [153, 155].

### PD-1

**Fig. 6:**
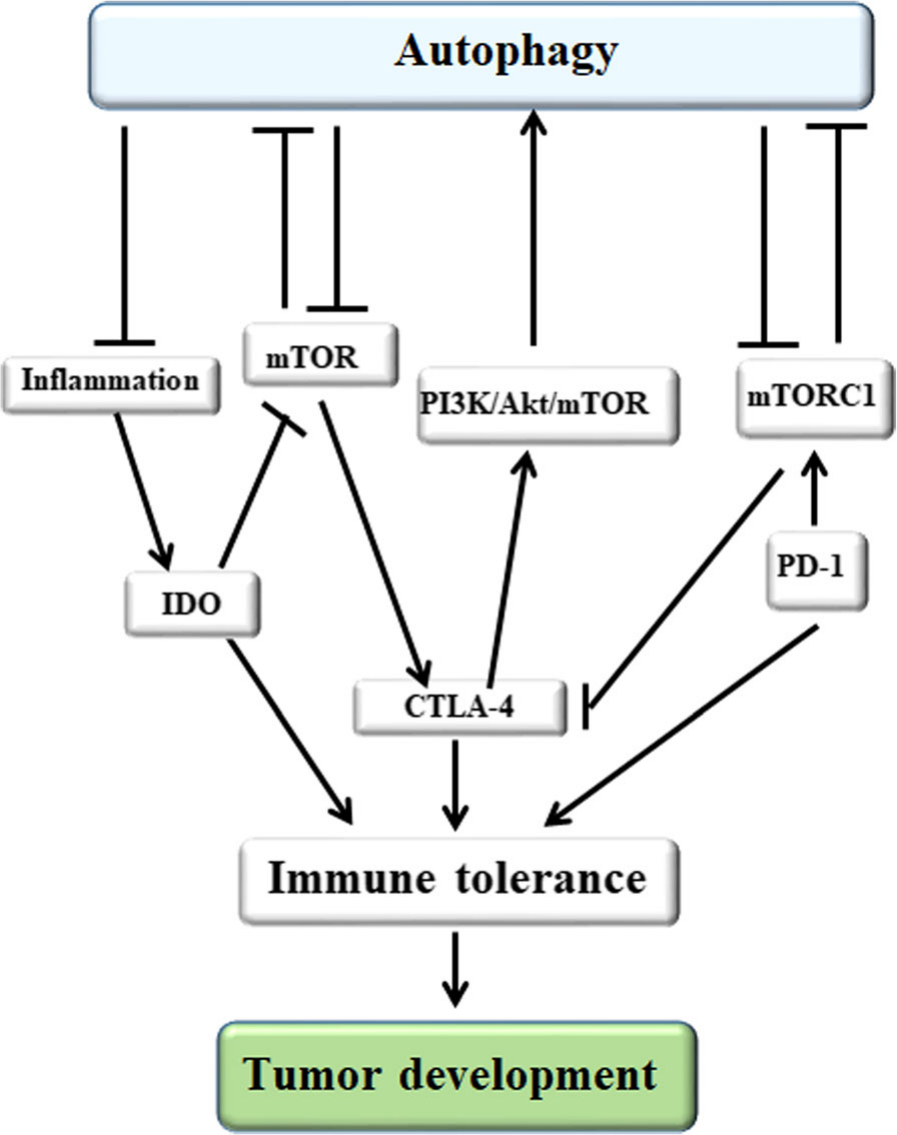
The relationship between autophagy and tumor immune tolerance. IDO is thought to potently inhibit effective anti-tumor immunity, drive immunologic tolerance and promote the development of tumor by suppressing cytotoxic T cell responses and inflammatory dendritic cell maturation, magnifying tolerogenic APCs and Tregs generation. IDO triggers autophagy by inhibiting a tryptophan sufficiency signal, resulting in the inhibition of mTOR, and autophagy can inhibit the inflammation-mediated expression IDO production by suppressing inflammation. CTLA-4 is an effective therapeutic target in tumor patients. Autophagy induction can improve anti-CTLA-4 curative effects, autophagy activation can restore the expression of CTLA-4 as well as suppressor function, CTLA4 engagement inhibits autophagy by constraining the transcription of LC-3β and the formation of autophagosomes. PD-1 acts as a T-cell inhibitory checkpoint molecule and suppresses anti-tumor immunity by developing a T-cell tolerance, inhibiting T cells proliferation, and hindering the recognition of tumor cells via interaction with PD-L1 on the surface of tumor cells, and tumor cell-intrinsic PD-L1 can suppress autophagy by activating mTORC1 signaling and inhibiting mTORC2 signaling

PD-1 acts as a T-cell inhibitory checkpoint molecule and suppresses anti-tumor immunity by developing a T-cell tolerance, inhibiting T cell proliferation, and hindering the recognition of tumor cells via interaction with PD-L1 on the surface of tumor cells. PD-L1/PD-1 engagement can induce autophagy in nearby T-cells due to the deprivation of nutrients [156]. Sigma1 inhibitor has been identified to induce degradation of PD-L1 and suppress the functional interaction of PD-1 and PD-L1 in a co-culture of T-cells and tumor cells via autophagy. Therefore, we believe that Sigma1 modulators can ameliorate the tumor immune microenvironment by acting on PD-L1/PD-1 blockade [157]. Recent reports have shown that blocking the PD-L1/PD-1 axis via anti-PD1 or anti-PD-L1 antibodies can trigger autophagy in tumor cells and be an attractive tumor immunotherapy when coupled with autophagy inhibitors [158]. Although clinical trials focus on blocking the PD-L1/PD-1 conjugation, there also exist severe consequences in the absence of PD1. For instance, *Mycobacterium tuberculosis*-infected PD-1−/− mice exhibit dramatically lower antigen-specific immune response. The numbers of T cells and B cells are reduced due to the increased numbers of Tregs and mesenchymal stem cells, and antigen-specific T cells may be defective. Moreover, because of their inability to proliferate, the capacity of these cells to consume cytokines is reduced, which results in enhanced Th1, Th2, and Th17 cytokines. In addition, *Mycobacterium tuberculosis*-infected PD-1−/− macrophages do not undergo autophagy to sustain homeostasis, which is proven by decreased autophagy-induced LC3β [159] (Fig. 6).

### CTLA-4

As an immune tolerance checkpoint, CTLA-4 is an effective therapeutic target in tumor patients. It was confirmed that in human melanomas, the expression of the key autophagosome component LC3-β and other autophagy activators are available to suppress primary resistance to CTLA-4 blockade through decreasing MAGE-A protein levels and blocking the MAGE-TRIM28 complex, which indicates that autophagy induction can improve anti-CTLA-4 curative effects [160]. Nevertheless, autophagy activation by 4-week rapamycin or other mTORC1 inhibitors treatment restore the expression of CTLA-4 as well as suppressor function and expands CD4 + CD25 + FOXP3+ Tregs in SLE patients [161]. Importantly, CTLA4 engagement significantly enhances activation of the PI3K/Akt/mTOR pathway and induces FoxO1 translocates to the nucleus, which in turn inhibits autophagy by constraining the transcription of LC-3β and the formation of autophagosomes [97].

In conclusion, IDO is thought to potently inhibit effective anti-tumor immunity, drive immunologic tolerance and promote the development of tumor. IDO triggers autophagy by inhibiting a tryptophan sufficiency signal, resulting in the inhibition of mTOR, and autophagy can inhibit the inflammation-mediated expression IDO production by suppressing inflammation. PD-1 acts as a T-cell inhibitory checkpoint molecule and suppresses anti-tumor immunity by developing a T-cell tolerance, inhibiting T cells proliferation, and hindering the recognition of tumor cells via interaction with PD-L1 on the surface of tumor cells, and tumor cell-intrinsic PD-L1 can suppress autophagy by activating mTORC1 signaling and inhibiting mTORC2 signaling. CTLA4, as an immune tolerance checkpoint, its engagement significantly prohibits autophagy by enhancing activation of the PI3K/Akt/mTOR pathway and promoting the translocation of FoxO1 from the nucleus, and autophagy activation can enhance CTLA-4 expression and restore the suppressor function. Therefore, autophagy is regulated by a series of tumor immune tolerance molecules and it also plays a key role in regulating tumor immune tolerance (Fig. 6).

## The applications of autophagy for tumor immunotherapy

The immune system plays a dominant role in tumor treatment by identifying and killing tumor cells during different stages of tumor development. Accumulating studies show that autophagy could up-regulate and down-regulate the immune response by influencing cells and the release of cytokines, which has provided targets and enlightenment for tumor immunotherapy [44]. As a new generation of anti-tumor therapeutics, tumor immunotherapy plays a predominant role in suppressing tumor development and will continue to progress. So far, antibody targeting therapy synergy with autophagy has been frequently reported. For instance, in ovarian cancer models, MORAB-003 (farletuzumab), a humanized monoclonal antibody against folate receptor alpha (FRα), has displayed a notable anti-tumor effect through antibody-dependent cellular cytotoxicity by sustaining late-stage autophagy, and when protein and organelle turnover overwhelm the capacity of the cell, which contributes to type II programmed cell death or autophagic death [162, 163]. In addition, CD73 enzymes play a pivotal role in generating an immunosuppressed and pro-angiogenic niche to support tumor development. Pharmacological blockade of CD73 with MEDI9447 (an Anti-CD73 Ab) increases antigen presentation and autophagy, resulting in enhanced lymphocyte activation and a greater release of proinflammatory Th1 cytokines [164, 165].

### Autophagy enhances the effects of immunotherapy

Recently, therapies aiming at autophagy to enhance the immune responses and anti-tumor effects of immunotherapy have become the prospective strategies, with enhanced angtigen presentation and higher sensitivity to CTLs [65]. Radiotherapy and chemotherapy might provoke autophagy; therefore, combining immunotherapy with radiotherapy or chemotherapy produces better treatment effects. Radiation or chemotherapy-induced autophagy has been reported to redistribute mannose-6-phopsphate receptor (MPR) with its ligands to the autophagosomes via clathrin-coated vesicles. Low pH in autophagosomes leads to the release of the MPR cargo. Empty MPRs are transported back to the tumor cell surface. Receptors bind to granzyme B (GrzB, one such ligand of MPR) produced by activated CTLs, which renders tumor cells more susceptible to CTLs killing and potentiates the effect of immunotherapy [166, 167]. In addition, autophagy plays a role in antigen processing for MHCI and MHCII presentation. The semisynthetic vitamin E derivative alpha-tocopheryloxyacetic acid (α-TEA) can stimulate autophagy to strengthen MHCI cross-presentation of tumor antigens to antigen-special CD8+ T cells, which is viewed as an adjuvant strategy to improve immunotherapy by reinforcing anti-tumor immune responses [62, 63]. In atherosclerotic lesions, oxidized low-density lipoprotein (OxLDL) induces the phosphorylation of spleen tyrosine kinase (SYK), and activated SYK induces autophagy by enhancing ROS production and MAPK8/9 activity, which in turn results in the release of BECN1 from a BECN1-BCL2 complex and autophagosome formation. Moreover, SYK augments OxLDL-induced autophagy and MHCII expression in macrophages. The OxLDL-induced and SYK-mediated autophagy facilitates surface expression of MHCII and CD4+ T cells activation; thereby, SYK may enhance anti-tumor immunotherapy effects via a autophagy-mediated adaptive immune responses [168]. In recent studies, DCs-based vaccines have shown promising therapeutic effects in promoting tumor immunotherapy by boosting antigen presentation. For example, lactosylated N-Alkyl polyethylenimine coated superparamagnetic iron oxide (SPIO) nanoparticles-induced autophagy can enhance the vaccine functions of DCs by inducing DCs maturation [169]. Furthermore, Shikonin-induced autophagy can directly contribute to damage-associated molecular patterns (DAMPs) upregulation and DCs activation, DCs vaccine preparations need the pretreatment of CQ, which will enhance the anti-metastatic effect of shikonin [170]. Importantly, studies have found that DRibble-loaded DCs efficiently induce cross-reactive and antigen-specific T cells generation by enhancing DCs cross-presenting antigens on up-regulating MHC-I expression, the formation of DRibble is induced by short-lived proteins (SLiPs) and depends on ubiquitinated proteins and the SQSTM1/p62, both of which co-localize with LC3, and p62 is necessary for the delivery of ubiquitinated proteins to autophagosomes [171, 172]. Additionally, autophagy can also improve the efficacy of DNA vaccines by synthetizing intracellular vaccine-encoded tumor antigen [173]. SQSTM1/p62 is related to autophagy and can be chosen as a novel cancer antigen. Researchers have observed anti-tumor and anti-metastatic activity of p62-encoding DNA vaccines, which is a promising strategy for tumor immunotherapy [174].

### Autophagy attenuates the effects of immunotherapy

However, it has been reported that hypoxia-induced autophagy has attenuated the effects of immunotherapy by impairing CTLs-mediated tumor cell lysis associated with the hypoxia-dependent phosphorylation of STAT3 (pSTAT3). Activated STAT3 promotes tumor cell survival, proliferation, angiogenesis/metastasis and immune escape. The first mechanism is that the HIF-1α-dependent intrinsic signaling pathway is activated, which phosphorylates Src kinase in the Tyr416 residue (pSrc), leading to STAT3 phosphorylation of the Tyr705 residue. The second is that HIF-1α induces autophagy by upregulating BNIP3/BNIP3L expression and dissociating the BECN1-BCL2 complex, which results in degradation of the SQSTM1/p62 protein that transfers pSTAT3 to the UPS for accumulation in cells. When autophagy is blocked, p62 is accumulated and in turn accelerates the delivery of pSTAT3 to the UPS for selective degradation [175, 176]. Hypoxia-induced autophagy also degrades NK-derived GrzB and impairs NK-mediated killing, as accumulated HIF-2α transfers to the nucleus and induces the expression of the autophagy sensor ITPR1, leading to the impairment of NK-mediated killing and decreased immunotherapy effects [156, 165]. Many studies indicate that autophagy inhibition in tumors can be viewed as an approach to improve anti-tumor immunotherapies. HDIL-2 alone has been found to be an efficient immunotherapy method in an advanced murine metastatic liver tumor model. IL-2 inhibits tumor growth by enhancing immune cell proliferation and infiltration in the liver and spleen; however, the anti-tumor effects of HDIL-2 immunotherapy were significantly heightened when coupled with administration of autophagy inhibitor CQ [132]. Similarly, in renal cell carcinoma, CQ is also used to improve HDIL-2-mediated anti-tumor immunity by enhancing DCs, T-cells and NK cells and limiting ATP production through inhibition of oxidative phosphorylation and promotion of apoptosis [177]. Another selective PI3K inhibitor, 3-MA acts on Vps34 and PI3Kγ and significantly enhances IL-24-induced apoptosis in oral squamous cellcarcinomas (OSCC), which demonstrates the combination of autophagy inhibitors and IL-24 is a promising approach for tumor immunotherapy [178].

## Conclusion

**Fig. 7:**
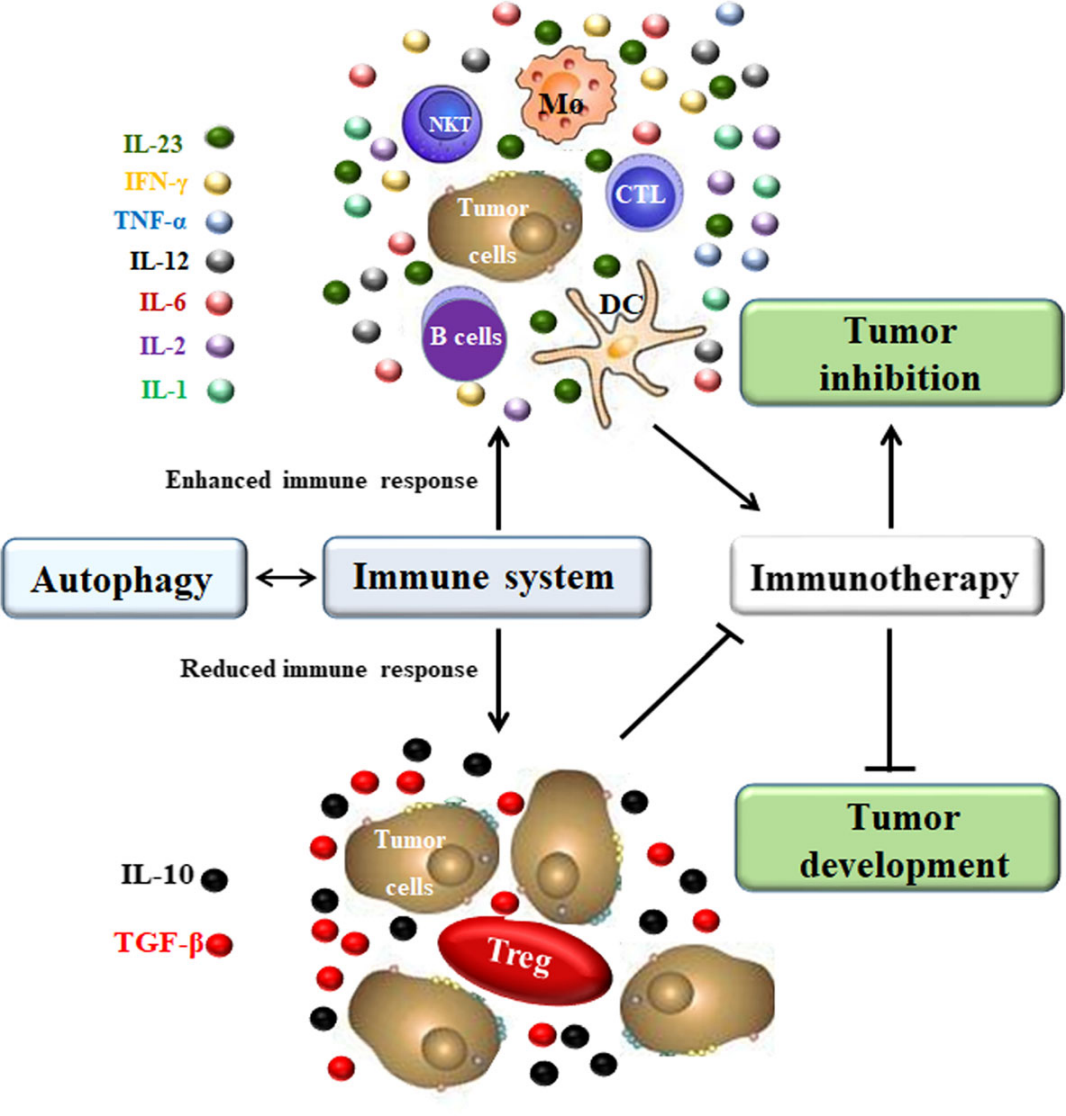
The applications of autophagy for tumor immunotherapy. There is a complicated interaction between autophagy and immune system. Autophagy can enhance immune response by ensuring the inhibitory action of CTL, B cell, Mø, NKT and DC on tumor cells and the release of immunoreactive cytokines, like IL-1, IL-2, IL-6, IL-12, IL-23, TNF-α and IFN-γ, resulting in enhanced anti-tumor immunotherapy effects and repressed tumor development. In addition, autophagy can also reduce immune response by recruiting immunosuppressive Tregs and promoting IL-10 and TGF-β production, contributing to attenuated anti-tumor immunotherapy effects and accelerated tumor development

Autophagy is required for the maintenance of metabolic and genetic homeostasis in eukaryotic organisms, which is involved with various ATG protein complexes regulated by several signaling pathways. Autophagy plays a dual role in tumor cell growth, which is dependent on the properties of the tumor and cell types. Therefore, when and how autophagy can be pro-survival and pro-death should be carefully interpreted in the future. In the tumor microenvironment, autophagy is an important regulator of immune responses by sustaining homeostasis, activation, and biological functions of immune cells. However, the autophagy-mediated regulation of the immune system might strengthen or attenuate the effects of immunotherapy (Fig. 7). Therefore, whether we should try to enhance or inhibit autophagy in anti-tumor immunotherapy remains to be explored. Many studies have demonstrated that the optimal combination of autophagy-based inducer or inhibitor with various therapeutic strategies, including chemotherapy, radiotherapy, immunotherapy, and gene therapy may be a more efficient approach by eliciting tumor cell death. Furthermore, many findings confirmed that both autophagy activators and inhibitors are being preclinical studies and potential to cure various tumors in the future (Tables 1 and 2). Nevertheless, only a few autophagy inhibitive agents are being applied to strengthen the anti-tumor effects of immunotherapy in preclinical studies (Table 3). Even though, studies about the application of autophagy inhibitors or enhancers alone in clinical treatment have not yet been published, importantly, several clinical trials have been shown that autophagy inhibitors hydroxychloroquine(HCQ) and CQ, autophagy activators aspirin (ASA) combined with other antineoplastic drugs significantly improve the therapeutic effect in tumors (Table 4). In the future, efforts should be focused on how to regulate autophagy to strengthen innate and adaptive immune responses and overcome anti-tumor immune resistance in immunotherapy for tumors.

**Table 1:** The application of autophagy activators in tumor therapy

Autophagy Activators	Tumor Types	Autophagy-Modulating Mechanism	Research Types
2-Methoxyestradiol	Osteosarcoma	Induce RNA-dependent protein kinase (PKR)-dependent autophagy [179]	Preclinical
Neferine	Neuroblastoma	Down-regulate focal adhesion kinase (FAK) and Beclin1-mediated autophagy [180]	Preclinical
Honokiol	Prostate cancer	Induce ROS-dependent autophagy [181]	Preclinical
ADIPOQ/adiponectin	Breast cancer	Stimulate STK11/LKB1-AMPK-ULK1-mediated autophagy [182]	Preclinical
2-amino-nicotinonitrile compound	Gastric tumors	Activate EGFR-mediated RAS-RAF1-MAP2K-MAPK1/3 signaling pathway [183]	Preclinical
MIR506	Pancreatic cancer	Mediate STAT3-BCL2-BECN1 signaling pathway [184]	Preclinical
Shikonin	Liver cancer	Promote the accumulation of reactive oxygen species and phospho-ERK [185]	Preclinical
Salidroside	Colorectal cancer	Suppress the PI3K/Akt/mTOR signaling pathways [186]	Preclinical
AZD8055	Head and neck squamous cell carcinoma (HNSCC)	Blocking mTORC1 and mTORC2 activation and inducing JNK activity [187]	Preclinical

**Table 2:** The application of autophagy inhibitors in tumor therapy

Autophagy Inhibitors	Tumor Types	Autophagy-Modulating Mechanism	Research Types
Quercetin	Cervical cancer	Inhibit LC-3 and beclin-1 activation [188]	Preclinical
Chloroquine	Bladder cancer	Prohibit lysosomal functions and autophagy [189]	Preclinical
SB202190	Colorectal Cancer	Cause cell cycle arrest and autophagic cell death [190]	Preclinical
Elaiophylin	Ovarian cancer	Block autophagic flux by attenuating lysosomal cathepsin activity [191]	Preclinical
Peiminine	Glioblastoma	Inhibit autophagy by inhibiting AMPK-ULK1 pathways [192]	Preclinical
U0126	Pancreatic Cancer	Induce apoptosis after autophagy inhibition [193]	Preclinical

**Table 3:** The application of autophagy inhibitors in tumor immunotherapy

Autophagy Inhibitors	Tumor Types	Autophagy-Modulating Mechanism	Research Types
Chloroquine	Renal cell carcinoma	Improve HDIL-2-mediated anti-tumor immunity by enhancing DCs, T-cells and NK cells [177]	Preclinical
3-methyladenine (3-MA)	Oral squamous cell Carcinomas	Significantly enhance IL-24-induced apoptosis [178]	Preclinical

**Table 4:** The translational application of autophagy inhibitors/ activators in tumor therapy

Drug Names	Autophagy Inhibitors/Activators	Combination Treatment	Tumor Types	Phase of Clinical Trials
Hydroxychloroquine (HCQ)	Inhibitor	Docetaxel	Metastatic Prostate Cancer	Phase2
HCQ	Inhibitor	Gemcitabine and Docetaxel	Recurrent or Refractory Osteosarcoma	Phase2
HCQ	Inhibitor	Dabrafenib and Trametinib	Melanoma	Phase2
HCQ	Inhibitor	Sirolimus or Vorinostat	Advanced Cancer	Phase1
HCQ	Inhibitor	Ixabepilone	Breast Cancer	Phase2
HCQ	Inhibitor	Mitoxantrone and Etoposid	Relapsed Acute Myelogenous Leukemia	Phase1
CQ	Inhibitor	Radiotherapy with daily temozolomide	Glioblastoma (GBM)	Phase1
aspirin (ASA)	Activator	Metformin (MET)	Colorectal Cancer	Phase2

* The data originated from: https://clinicaltrials.gov

## Abbreviations

3-MA: 3-methyladenine
AMPK: 5’ AMP-activated protein kinase
APC: Antigen-presenting cell
ASA: Aspirin
ATG: Autophagy-related genes
CQ: Chloroquine
CSF1: Colony-stimulating factor 1
CTL: Cytotoxic T-lymphocyte
DAMPs: Damage-associated molecular patterns
Dapk: Death-associated protein kinase
DEDD: Domain-containing DNA-binding protein
DRAM1: DNA damage-regulated autophagy modulator 1
Dribbles: Tumor-derived autophagosomes
EMT: Epithelial-to-mesenchymal transition
FasL: Fas ligand
fMLP: Formyl-Met-Leu-Phe
FRα: Folate receptor alpha
GCN2: General control nonderepressible 2
G-CSF: Granulocyte colony-stimulating factor
GM-CSF: Granulocyte macrophage colony-stimulating factor
HCQ: Hydroxychloroquine
HDIL-2: High-dose IL-2
HIF-1α: Hypoxia-inducible factor 1α
HMGB1: High mobility group box 1
IDO: Indoleamine 2, 3 dioxygenase
IP3Rs: Inositol 1, 4, 5-trisphosphate receptors
IRF8: Interferon regulatory factor 8
ITPR1: Inositol 1, 4, 5-triphosphate receptor, type I
JNK: c-Jun N-terminal kinases
LAP: Liver-enriched activator protein
LPS: Ligand lipopolysaccharide
MDSCs: Myeloid-derived suppressor cells
MMP9: Matrix metalloproteinase-9
MPR: Mannose-6-phopsphate receptor
Mtb: *Mycobacterium tuberculosis*
mTORC1: Mammalian target of rapamycin 1
NADPH: Nicotinamide adenine dinucleotide phosphate
NETs: Neutrophil extracellular traps
NF-κB: Nuclear factor kappa B
NLRs: Nucleotide oligomerization domain (NOD)-like receptors
NRF2: Nuclear factor erythroid 2–related factor 2
OSM: Oncostatin M
OxLDL: Oxidized low-density lipoprotein
p38-MAPK: Mitogen-activated protein kinase
Parkin: PARK2
PI3K: Phospholipids inositol triphosphate-kinase
PTEN: Phosphatase and tensin homolog deleted from chromosome ten
ROS: Reactive oxygen species
SLiPs: Short-lived proteins
SPIO: Superparamagnetic iron oxide
SQSTM1: Sequestosome 1
STAT1: Signal transducer and activator of transcription 1
TAM: Tumor-associated macrophage
TAMs: Tumor-associated macrophages
TLRs: Toll-like receptors
TRAIL: Tumor necrosis-like apoptosis-inducing ligand
TRIF: Toll-IL-1 receptor (TIR) domain-containing adapter-inducing IFN
Trp: Tryptophan
UPS: Ubiquitin proteasome system
VMP1: Vacuole membrane protein 1

## References

[1] Viry E, Paggetti J, Baginska J (2014). Autophagy: an adaptive metabolic response to stress shaping the antitumor immunity. Biochem Pharmacol.

[2] Wang S, Xia P, Rehm M (2015). Autophagy and cell reprogramming. Cellular and molecular life sciences. Cell Mol Life Sci.

[3] Feng Y, He D, Yao Z (2014). The machinery of macroautophagy. Cell Res.

[4] Fitzwalter BE, Thorburn A (2015). Recent insights into cell death and autophagy. FEBS J.

[5] Honscheid P, Datta K, Muders MH (2014). Autophagy: detection, regulation and its role in cancer and therapy response. Int J Radiat Biol.

[6] Plaza-Zabala A, Sierra-Torre V, Sierra A. Autophagy and microglia: novel Partners in Neurodegeneration and Aging. Int J Mol Sci. 2017;18(3). 10.3390/ijms18030598 PMC5372614 28282924

[7] Rockel JS, Kapoor M (2016). Autophagy: controlling cell fate in rheumatic diseases. Nat Rev Rheumatol.

[8] Wang D, Yu W, Liu Y (2017). Roles of autophagy in ischemic heart diseases and the modulatory effects of Chinese herbal medicine. AM J Chinese Med.

[9] Schreiber KH, Ortiz D, Academia EC (2015). Rapamycin-mediated mTORC2 inhibition is determined by the relative expression of FK506-binding proteins. Aging Cell.

[10] Laplante M, Sabatini DM (2012). mTOR signaling in growth control and disease. Cell.

[11] Lv Q, Hua F, Hu ZW (2012). DEDD, a novel tumor repressor, reverses epithelial-mesenchymal transition by activating selective autophagy. Autophagy.

[12] Catalano M, D'Alessandro G, Lepore F (2015). Autophagy induction impairs migration and invasion by reversing EMT in glioblastoma cells. Mol Oncol.

[13] Cheong H (2015). Integrating autophagy and metabolism in cancer. Arch Pharm Res.

[14] Harris J (2011). Autophagy and cytokines. Cytokine.

[15] Li CJ, Liao WT, Wu MY, et al. New insights into the role of autophagy in tumor immune microenvironment. Int J Mol Sci. 2017;18(7). 10.3390/ijms18071566 PMC5536054 28753959

[16] Mizushima N, Yoshimori T, Ohsumi Y (2011). The role of Atg proteins in autophagosome formation. Annu Rev Cell Dev Biol.

[17] Suzuki SW, Yamamoto H, Oikawa Y (2015). Atg13 HORMA domain recruits Atg9 vesicles during autophagosome formation. Proc Natl Acad Sci U S A.

[18] Jain MV, Paczulla AM, Klonisch T (2013). Interconnections between apoptotic, autophagic and necrotic pathways: implications for cancer therapy development. J Cell Mol Med.

[19] Czarny P, Pawlowska E, Bialkowska-Warzecha J (2015). Autophagy in DNA damage response. Int J Mol Sci.

[20] Zhou ZW, Li XX, He ZX (2015). Induction of apoptosis and autophagy via sirtuin1- and PI3K/Akt/mTOR-mediated pathways by plumbagin in human prostate cancer cells. Drug Des, Devel Ther.

[21] Cagnol S, Chambard JC (2010). ERK and cell death: mechanisms of ERK-induced cell death--apoptosis, autophagy and senescence. FEBS J.

[22] Samatar AA, Poulikakos PI (2014). Targeting RAS-ERK signalling in cancer: promises and challenges. Nat Rev Drug Discov.

[23] Zhou YY, Li Y, Jiang WQ, et al. MAPK/JNK signalling: a potential autophagy regulation pathway. Biosci Rep. 2015;35(3). 10.1042/BSR20140141 PMC4613668 26182361

[24] Zhong L, Shu W, Dai W, et al. Reactive oxygen species-mediated c-Jun NH2-terminal kinase activation contributes to hepatitis B virus X protein-induced autophagy via regulation of the Beclin-1/Bcl-2 interaction. J Virol. 2017;91(15). 10.1128/JVI.00001-17 PMC5512237 28515304

[25] Yang J, Yao S (2015). JNK-Bcl-2/Bcl-xL-Bax/Bak pathway mediates the crosstalk between Matrine-induced autophagy and apoptosis via interplay with Beclin 1. Int J Mol Sci.

[26] Kania E, Roest G, Vervliet T (2017). IP3 receptor-mediated calcium signaling and its role in autophagy in cancer. Front Oncol.

[27] Fedorenko OA, Popugaeva E, Enomoto M (2014). Intracellular calcium channels: inositol-1,4,5-trisphosphate receptors. Eur J Pharmacol.

[28] Van Petegem F (2015). Ryanodine receptors: allosteric ion channel giants. J Mol Biol.

[29] Wang Q, Huang L, Yue J (2017). Oxidative stress activates the TRPM2-ca(2+)-CaMKII-ROS signaling loop to induce cell death in cancer cells. Biochim Biophys Acta.

[30] Sahni S, Merlot AM, Krishan S (2014). Gene of the month: BECN1. J Clin Pathol.

[31] Laddha SV, Ganesan S, Chan CS (2014). Mutational landscape of the essential autophagy gene BECN1 in human cancers. Mol Cancer Res.

[32] Gong Y, Zack TI, Morris LG (2014). Pan-cancer genetic analysis identifies PARK2 as a master regulator of G1/S cyclins. Nat Genet.

[33] Moscat J, Diaz-Meco MT (2012). p62: a versatile multitasker takes on cancer. Trends Biochem Sci.

[34] Sun K, Xu L, Jing Y (2017). Autophagy-deficient Kupffer cells promote tumorigenesis by enhancing mtROS-NF-kappaB-IL1alpha/beta-dependent inflammation and fibrosis during the preneoplastic stage of hepatocarcinogenesis. Cancer Lett.

[35] Yang A, Rajeshkumar NV, Wang X (2014). Autophagy is critical for pancreatic tumor growth and progression in tumors with p53 alterations. Cancer Discov.

[36] Dimberg LY, Anderson CK, Camidge R (2013). On the TRAIL to successful cancer therapy? Predicting and counteracting resistance against TRAIL-based therapeutics. Oncogene.

[37] Thorburn A, Thamm DH, Gustafson DL (2014). Autophagy and cancer therapy. Mol Pharmacol.

[38] Lee DH, Nam YJ, Kim YJ (2014). Rotundarpene prevents TRAIL-induced apoptosis in human keratinocytes by suppressing the caspase-8- and bid-pathways and the mitochondrial pathway. Naunyn Schmiedeberg's Arch Pharmacol.

[39] White E, Mehnert JM, Chan CS (2015). Autophagy, metabolism, and Cancer. Clin Cancer Res.

[40] Guo JY, Xia B, White E (2013). Autophagy-mediated tumor promotion. Cell.

[41] Rosenfeldt MT, O'Prey J, Morton JP (2013). p53 status determines the role of autophagy in pancreatic tumour development. Nature.

[42] Rao S, Tortola L, Perlot T (2014). A dual role for autophagy in a murine model of lung cancer. Nat Commun.

[43] Li YY, Lam SK, Mak JC (2013). Erlotinib-induced autophagy in epidermal growth factor receptor mutated non-small cell lung cancer. Lung Cancer.

[44] Pan H, Chen L, Xu Y (2016). Autophagy-associated immune responses and cancer immunotherapy. Oncotarget.

[45] Fang L, Wu HM, Ding PS (2014). TLR2 mediates phagocytosis and autophagy through JNK signaling pathway in Staphylococcus aureus-stimulated RAW264.7 cells. Cell Signal.

[46] Lu Z, Xie D, Chen Y (2017). TLR2 mediates autophagy through ERK signaling pathway in mycoplasma gallisepticum-infected RAW264.7 cells. Mol Immunol.

[47] Delgado MA, Elmaoued RA, Davis AS (2008). Toll-like receptors control autophagy. EMBO J.

[48] Xu Y, Jagannath C, Liu XD (2007). Toll-like receptor 4 is a sensor for autophagy associated with innate immunity. Immunity.

[49] Zhan Z, Xie X, Cao H (2014). Autophagy facilitates TLR4- and TLR3-triggered migration and invasion of lung cancer cells through the promotion of TRAF6 ubiquitination. Autophagy.

[50] van der Vaart M, Korbee CJ, Lamers GE, Tengeler AC, Hosseini R, Haks MC, Ottenhoff TH, Spaink HP, Meijer AH (2014). The DNA damage-regulated autophagy modulator DRAM1 links mycobacterial recognition via TLR-MYD88 to autophagic defense. Cell Host Microbe.

[51] Lupfer C, Kanneganti TD (2013). The expanding role of NLRs in antiviral immunity. Immunol Rev.

[52] Carneiro LA, Travassos LH (2013). The interplay between NLRs and autophagy in immunity and inflammation. Front Immunol.

[53] Sorbara MT, Ellison LK, Ramjeet M (2013). The protein ATG16L1 suppresses inflammatory cytokines induced by the intracellular sensors Nod1 and Nod2 in an autophagy-independent manner. Immunity.

[54] Oh JE, Lee HK (2013). Autophagy as an innate immune modulator. Immune Netw.

[55] Selvanantham T, Escalante NK, Cruz Tleugabulova M (2013). Nod1 and Nod2 enhance TLR-mediated invariant NKT cell activation during bacterial infection. J Immunol.

[56] Ozbayer C, Kurt H, Bayramoglu A (2015). The role of NOD1/CARD4 and NOD2/CARD15 genetic variations in lung cancer risk. Inflamm Res.

[57] Gupta M, Shin DM, Ramakrishna L (2015). IRF8 directs stress-induced autophagy in macrophages and promotes clearance of listeria monocytogenes. Nat Commun.

[58] Zhong Z, Sanchez-Lopez E, Karin M (2016). Autophagy, inflammation, and immunity: a troika governing Cancer and its treatment. Cell.

[59] Randow F, Munz C (2012). Autophagy in the regulation of pathogen replication and adaptive immunity. Trends Immunol.

[60] Munz C (2016). Autophagy beyond intracellular MHC class II antigen presentation. Trends Immunol.

[61] Saini NK, Baena A, Ng TW (2016). Suppression of autophagy and antigen presentation by mycobacterium tuberculosis PE_PGRS47. Nat Microbiol.

[62] Li Y, Hahn T, Garrison K (2012). The vitamin E analogue alpha-TEA stimulates tumor autophagy and enhances antigen cross-presentation. Cancer Res.

[63] Hahn T, Akporiaye ET (2013). Alpha-TEA as a stimulator of tumor autophagy and enhancer of antigen cross-presentation. Autophagy.

[64] Sena LA, Li S, Jairaman A (2013). Mitochondria are required for antigen-specific T cell activation through reactive oxygen species signaling. Immunity.

[65] Kim J, Kundu M, Viollet B (2011). AMPK and mTOR regulate autophagy through direct phosphorylation of Ulk1. Nat Cell Biol.

[66] Botbol Y, Patel B, Macian F (2015). Common gamma-chain cytokine signaling is required for macroautophagy induction during CD4+ T-cell activation. Autophagy.

[67] Hubbard VM, Valdor R, Patel B (2010). Macroautophagy regulates energy metabolism during effector T cell activation. J Immunol.

[68] Jia W, He MX, McLeod IX (2015). Autophagy regulates T lymphocyte proliferation through selective degradation of the cell-cycle inhibitor CDKN1B/p27Kip1. Autophagy.

[69] Pua HH, Guo J, Komatsu M (2009). Autophagy is essential for mitochondrial clearance in mature T lymphocytes. J Immunol.

[70] Kovacs JR, Li C, Yang Q (2012). Autophagy promotes T-cell survival through degradation of proteins of the cell death machinery. Cell Death Differ.

[71] Sowell RT, Rogozinska M, Nelson CE (2014). Cutting edge: generation of effector cells that localize to mucosal tissues and form resident memory CD8 T cells is controlled by mTOR. J Immunol.

[72] Bronietzki AW, Schuster M, Schmitz I (2015). Autophagy in T-cell development, activation and differentiation. Immunol Cell Biol.

[73] Xu X, Araki K, Li S (2014). Autophagy is essential for effector CD8(+) T cell survival and memory formation. Nat Immunol.

[74] Michalek RD, Gerriets VA, Jacobs SR (2011). Cutting edge: distinct glycolytic and lipid oxidative metabolic programs are essential for effector and regulatory CD4+ T cell subsets. J Immunol.

[75] Delgoffe GM, Pollizzi KN, Waickman AT (2011). The kinase mTOR regulates the differentiation of helper T cells through the selective activation of signaling by mTORC1 and mTORC2. Nat Immunol.

[76] Shi LZ, Wang R, Huang G (2011). HIF1alpha-dependent glycolytic pathway orchestrates a metabolic checkpoint for the differentiation of TH17 and Treg cells. J Exp Med.

[77] Garg AD, Dudek AM, Agostinis P (2013). Autophagy-dependent suppression of cancer immunogenicity and effector mechanisms of innate and adaptive immunity. Oncoimmunology..

[78] Matsuzawa T, Kim BH, Shenoy AR (2012). IFN-gamma elicits macrophage autophagy via the p38 MAPK signaling pathway. J Immunol.

[79] Kumar P (2017). IFNgamma-producing CD4(+) T lymphocytes: the double-edged swords in tuberculosis. Clin Transl Med.

[80] Rovetta AI, Pena D, Hernandez Del Pino RE (2014). IFNG-mediated immune responses enhance autophagy against mycobacterium tuberculosis antigens in patients with active tuberculosis. Autophagy.

[81] Ghadimi D, de Vrese M, Heller KJ (2010). Lactic acid bacteria enhance autophagic ability of mononuclear phagocytes by increasing Th1 autophagy-promoting cytokine (IFN-gamma) and nitric oxide (NO) levels and reducing Th2 autophagy-restraining cytokines (IL-4 and IL-13) in response to mycobacterium tuberculosis antigen. Int Immunopharmacol.

[82] Li WL, Xiong LX, Shi XY (2016). IKKbeta/NFkappaBp65 activated by interleukin-13 targets the autophagy-related genes LC3B and beclin 1 in fibroblasts co-cultured with breast cancer cells. Exp Ther Med.

[83] Xia F, Deng C, Jiang Y (2018). IL4 (interleukin 4) induces autophagy in B cells leading to exacerbated asthma. Autophagy.

[84] Wang S, Xia P, Huang G (2016). FoxO1-mediated autophagy is required for NK cell development and innate immunity. Nat Commun.

[85] Salio M, Puleston DJ, Mathan TS (2014). Essential role for autophagy during invariant NKT cell development. Proc Natl Acad Sci U S A.

[86] Pei B, Zhao M, Miller BC (2015). Invariant NKT cells require autophagy to coordinate proliferation and survival signals during differentiation. J Immunol.

[87] Buchser WJ, Laskow TC, Pavlik PJ (2012). Cell-mediated autophagy promotes cancer cell survival. Cancer Res.

[88] Wei J, Long L, Yang K (2016). Autophagy enforces functional integrity of regulatory T cells by coupling environmental cues and metabolic homeostasis. Nat Immunol.

[89] Zeng H, Yang K, Cloer C (2013). mTORC1 couples immune signals and metabolic programming to establish T(reg)-cell function. Nature.

[90] Rao S, Yang H, Penninger JM (2014). Autophagy in non-small cell lung carcinogenesis: a positive regulator of antitumor immunosurveillance. Autophagy.

[91] Ren T, Dong W, Takahashi Y (2012). HTLV-2 tax immortalizes human CD4+ memory T lymphocytes by oncogenic activation and dysregulation of autophagy. J Biol Chem.

[92] Chen L, Liu D, Zhang Y (2015). Foxp3-dependent transformation of human primary CD4+ T lymphocytes by the retroviral protein tax. Biochem Bioph Res Co..

[93] Parekh VV, Wu L, Boyd KL (2013). Impaired autophagy, defective T cell homeostasis, and a wasting syndrome in mice with a T cell-specific deletion of Vps34. J Immunol.

[94] Arnold J, Murera D, Arbogast F (2016). Autophagy is dispensable for B-cell development but essential for humoral autoimmune responses. Cell Death Differ.

[95] Zhou M, Li W, Wen Z (2015). Macrophages enhance tumor-derived autophagosomes (DRibbles)-induced B cells activation by TLR4/MyD88 and CD40/CD40L. Exp Cell Res.

[96] Fribourg M, Ni J, Nina Papavasiliou F (2018). Allospecific memory B cell responses are dependent on autophagy. Am J Transplant.

[97] Alissafi T, Banos A, Boon L (2017). Tregs restrain dendritic cell autophagy to ameliorate autoimmunity. J Clin Invest.

[98] Liu E, Van Grol J, Subauste CS (2015). Atg5 but not Atg7 in dendritic cells enhances IL-2 and IFN-gamma production by toxoplasma gondii-reactive CD4+ T cells. Microbes Infect.

[99] Seto S, Tsujimura K, Horii T (2013). Autophagy adaptor protein p62/SQSTM1 and autophagy-related gene Atg5 mediate autophagosome formation in response to mycobacterium tuberculosis infection in dendritic cells. PLoS One.

[100] Lee HK, Mattei LM, Steinberg BE (2010). In vivo requirement for Atg5 in antigen presentation by dendritic cells. Immunity.

[101] Chen P, Cescon M, Bonaldo P (2014). Autophagy-mediated regulation of macrophages and its applications for cancer. Autophagy.

[102] Jacquel A, Obba S, Solary E (2012). Proper macrophagic differentiation requires both autophagy and caspase activation. Autophagy.

[103] Zhang Y, Morgan MJ, Chen K (2012). Induction of autophagy is essential for monocyte-macrophage differentiation. Blood.

[104] Mantovani A, Biswas SK, Galdiero MR (2013). Macrophage plasticity and polarization in tissue repair and remodelling. J Pathol.

[105] Liu K, Zhao E, Ilyas G (2015). Impaired macrophage autophagy increases the immune response in obese mice by promoting proinflammatory macrophage polarization. Autophagy.

[106] Li N, Qin J, Lan L (2015). PTEN inhibits macrophage polarization from M1 to M2 through CCL2 and VEGF-A reduction and NHERF-1 synergism. Cancer Biol Ther.

[107] Rozman S, Yousefi S, Oberson K (2015). The generation of neutrophils in the bone marrow is controlled by autophagy. Cell Death Differ.

[108] Bhattacharya A, Wei Q, Shin JN (2015). Autophagy is required for neutrophil-mediated inflammation. Cell Rep.

[109] Itakura A, McCarty OJ (2013). Pivotal role for the mTOR pathway in the formation of neutrophil extracellular traps via regulation of autophagy. Am J Physiol Cell Physiol.

[110] Li XF, Chen DP, Ouyang FZ (2015). Increased autophagy sustains the survival and pro-tumourigenic effects of neutrophils in human hepatocellular carcinoma. J Hepatol.

[111] Yang X, Yu DD, Yan F (2015). The role of autophagy induced by tumor microenvironment in different cells and stages of cancer. Cell Biosci.

[112] Parker KH, Horn LA, Ostrand-Rosenberg S (2016). High-mobility group box protein 1 promotes the survival of myeloid-derived suppressor cells by inducing autophagy. J Leukoc Biol.

[113] Li W, Tanikawa T, Kryczek I (2018). Aerobic glycolysis controls myeloid-derived suppressor cells and tumor immunity via a specific CEBPB isoform in triple-negative breast Cancer. Cell Metab.

[114] De Veirman K, Menu E, Maes K (2018). Myeloid-derived suppressor cells induce multiple myeloma cell survival by activating the AMPK pathway. Cancer Lett.

[115] Schauer IG, Zhang J, Xing Z (2013). Interleukin-1beta promotes ovarian tumorigenesis through a p53/NF-kappaB-mediated inflammatory response in stromal fibroblasts. Neoplasia.

[116] Castillo EF, Dekonenko A, Arko-Mensah J (2012). Autophagy protects against active tuberculosis by suppressing bacterial burden and inflammation. Proc Natl Acad Sci U S A.

[117] Jiang S, Dupont N, Castillo EF (2013). Secretory versus degradative autophagy: unconventional secretion of inflammatory mediators. J Innate Immun.

[118] Lappas M (2014). Caspase-1 activation is increased with human labour in foetal membranes and myometrium and mediates infection-induced interleukin-1beta secretion. Am J Reprod Immunol.

[119] Peral de Castro C, Jones SA, Ni Cheallaigh C (2012). Autophagy regulates IL-23 secretion and innate T cell responses through effects on IL-1 secretion. J Immunol.

[120] Warr MR, Binnewies M, Flach J (2013). FOXO3A directs a protective autophagy program in haematopoietic stem cells. Nature.

[121] Schmeisser H, Bekisz J, Zoon KC (2014). New function of type I IFN: induction of autophagy. J Interf Cytokine Res.

[122] Tu SP, Quante M, Bhagat G (2011). IFN-gamma inhibits gastric carcinogenesis by inducing epithelial cell autophagy and T-cell apoptosis. Cancer Res.

[123] Sharma G, Dutta RK, Khan MA (2014). IL-27 inhibits IFN-gamma induced autophagy by concomitant induction of JAK/PI3 K/Akt/mTOR cascade and up-regulation of mcl-1 in mycobacterium tuberculosis H37Rv infected macrophages. Int J Biochem Cell Biol.

[124] Cho SH, Oh SY, Lane AP (2014). Regulation of nasal airway homeostasis and inflammation in mice by SHP-1 and Th2/Th1 signaling pathways. PLoS One.

[125] Li X, Li Y, Fang S (2017). Downregulation of autophagy-related gene ATG5 and GABARAP expression by IFN-lambda1 contributes to its anti-HCV activity in human hepatoma cells. Antivir Res.

[126] Qin B, Zhou Z, He J (2015). IL-6 inhibits starvation-induced autophagy via the STAT3/Bcl-2 signaling pathway. Sci Rep.

[127] Dutta RK, Kathania M, Raje M (2012). IL-6 inhibits IFN-gamma induced autophagy in mycobacterium tuberculosis H37Rv infected macrophages. Int J Biochem Cell Biol.

[128] Linnemann AK, Blumer J, Marasco MR, Battiola TJ, Umhoefer HM, Han JY, Lamming DW, Davis DB (2017). Interleukin 6 protects pancreatic beta cells from apoptosis by stimulation of autophagy. FASEB J.

[129] Pettersen K, Andersen S, Degen S (2017). Cancer cachexia associates with a systemic autophagy-inducing activity mimicked by cancer cell-derived IL-6 trans-signaling. Sci Rep.

[130] Luo MX, Wong SH, Chan MT (2015). Autophagy mediates HBx-induced nuclear factor-kappaB activation and release of IL-6, IL-8, and CXCL2 in hepatocytes. J Cell Physiol.

[131] Kang R, Tang D, Lotze MT (2013). Autophagy is required for IL-2-mediated fibroblast growth. Exp Cell Res.

[132] Liang X, De Vera ME, Buchser WJ (2012). Inhibiting systemic autophagy during interleukin 2 immunotherapy promotes long-term tumor regression. Cancer Res.

[133] Li ML, Xu YZ, Lu WJ (2018). Chloroquine potentiates the anticancer effect of sunitinib on renal cell carcinoma by inhibiting autophagy and inducing apoptosis. Oncol Lett.

[134] Lin Y, Kuang W, Wu B (2017). IL-12 induces autophagy in human breast cancer cells through AMPK and the PI3K/Akt pathway. Mol Med Rep.

[135] Santarelli R, Gonnella R, Di Giovenale G (2014). STAT3 activation by KSHV correlates with IL-10, IL-6 and IL-23 release and an autophagic block in dendritic cells. Sci Rep.

[136] Park HJ, Lee SJ, Kim SH (2011). IL-10 inhibits the starvation induced autophagy in macrophages via class I phosphatidylinositol 3-kinase (PI3K) pathway. Mol Immunol.

[137] Kishore R, Krishnamurthy P, Garikipati VN (2015). Interleukin-10 inhibits chronic angiotensin II-induced pathological autophagy. J Mol Cell Cardiol.

[138] Martin C, Espaillat MP, Santiago-Schwarz F (2015). IL-10 restricts dendritic cell (DC) growth at the monocyte-to-monocyte-derived DC interface by disrupting anti-apoptotic and cytoprotective autophagic molecular machinery. Immunol Res.

[139] Zhao J, Sun Y, Shi P (2015). Celastrol ameliorates experimental colitis in IL-10 deficient mice via the up-regulation of autophagy. Int Immunopharmacol.

[140] Qi GM, Jia LX, Li YL (2014). Adiponectin suppresses angiotensin II-induced inflammation and cardiac fibrosis through activation of macrophage autophagy. Endocrinology.

[141] Wang H, Wang Y, Li D (2015). VEGF inhibits the inflammation in spinal cord injury through activation of autophagy. Biochem Biophys Res Commun.

[142] Wang MX, Cheng XY, Jin M (2015). TNF compromises lysosome acidification and reduces alpha-synuclein degradation via autophagy in dopaminergic cells. Exp Neurol.

[143] Ullio C, Brunk UT, Urani C (2015). Autophagy of metallothioneins prevents TNF-induced oxidative stress and toxicity in hepatoma cells. Autophagy.

[144] Pun NT, Subedi A, Kim MJ (2015). Globular adiponectin causes tolerance to LPS-induced TNF-alpha expression via autophagy induction in RAW 264.7 macrophages: involvement of SIRT1/FoxO3A Axis. PLoS One.

[145] Wilson EB, El-Jawhari JJ, Neilson AL (2011). Human tumour immune evasion via TGF-beta blocks NK cell activation but not survival allowing therapeutic restoration of anti-tumour activity. PLoS One.

[146] Ding Y, Kim S, Lee SY (2014). Autophagy regulates TGF-beta expression and suppresses kidney fibrosis induced by unilateral ureteral obstruction. J Am Soc Nephrol.

[147] Zhang C, Zhang X, Xu R (2017). TGF-beta2 initiates autophagy via Smad and non-Smad pathway to promote glioma cells’ invasion. J Exp Clin Cancer Res.

[148] Suzuki HI, Kiyono K, Miyazono K (2010). Regulation of autophagy by transforming growth factor-beta (TGF-beta) signaling. Autophagy.

[149] Zheng T, Xu C, Mao C (2018). Increased Interleukin-23 in Hashimoto's thyroiditis disease induces autophagy suppression and reactive oxygen species accumulation. Front Immunol.

[150] McGaha TL, Huang L, Lemos H (2012). Amino acid catabolism: a pivotal regulator of innate and adaptive immunity. Immunol Rev.

[151] Folgiero V, Miele E, Carai A (2016). IDO1 involvement in mTOR pathway: a molecular mechanism of resistance to mTOR targeting in medulloblastoma. Oncotarget.

[152] Mahoney KM, Rennert PD, Freeman GJ (2015). Combination cancer immunotherapy and new immunomodulatory targets. Nat Rev Drug Discov.

[153] Metz R, Rust S, Duhadaway JB (2012). IDO inhibits a tryptophan sufficiency signal that stimulates mTOR: a novel IDO effector pathway targeted by D-1-methyl-tryptophan. Oncoimmunology..

[154] McGaha TL (2015). IDO-GCN2 and autophagy in inflammation. Oncotarget.

[155] Gupta S, Manicassamy S, Vasu C (2008). Differential requirement of PKC-theta in the development and function of natural regulatory T cells. Mol Immunol.

[156] Robainas M, Otano R, Bueno S (2017). Understanding the role of PD-L1/PD1 pathway blockade and autophagy in cancer therapy. Onco Targets Ther.

[157] Maher CM, Thomas JD, Haas DA (2018). Small-molecule Sigma1 modulator induces Autophagic degradation of PD-L1. Mol Cancer Res.

[158] Clark CA, Gupta HB, Curiel TJ (2017). Tumor cell-intrinsic CD274/PD-L1: a novel metabolic balancing act with clinical potential. Autophagy.

[159] Tousif S, Singh Y, Prasad DV (2011). T cells from programmed Death-1 deficient mice respond poorly to mycobacterium tuberculosis infection. PLoS One.

[160] Shukla SA, Bachireddy P, Schilling B (2018). Cancer-germline antigen expression discriminates clinical outcome to CTLA-4 blockade. Cell.

[161] Kato H, Perl A (2018). Blockade of Treg cell differentiation and function by the Interleukin-21-mechanistic target of rapamycin Axis via suppression of autophagy in patients with systemic lupus erythematosus. Arthritis Rheumatol.

[162] Wen Y, Graybill WS, Previs RA (2015). Immunotherapy targeting folate receptor induces cell death associated with autophagy in ovarian cancer. Clin Cancer Res.

[163] Wen Y, Zand B, Ozpolat B (2014). Antagonism of tumoral prolactin receptor promotes autophagy-related cell death. Cell Rep.

[164] Antonioli L, Blandizzi C, Malavasi F (2016). Anti-CD73 immunotherapy: a viable way to reprogram the tumor microenvironment. Oncoimmunology.

[165] Hay CM, Sult E, Huang Q (2016). Targeting CD73 in the tumor microenvironment with MEDI9447. Oncoimmunology..

[166] Kim S, Ramakrishnan R, Lavilla-Alonso S (2014). Radiation-induced autophagy potentiates immunotherapy of cancer via up-regulation of mannose 6-phosphate receptor on tumor cells in mice. Cancer Immunol Immunother.

[167] Ramakrishnan R, Huang C, Cho HI (2012). Autophagy induced by conventional chemotherapy mediates tumor cell sensitivity to immunotherapy. Cancer Res.

[168] Choi SH, Gonen A, Diehl CJ (2015). SYK regulates macrophage MHC-II expression via activation of autophagy in response to oxidized LDL. Autophagy.

[169] Shen T, Zhu W, Yang L (2018). Lactosylated N-alkyl polyethylenimine coated iron oxide nanoparticles induced autophagy in mouse dendritic cells. Regen Biomater.

[170] Lin SY, Hsieh SY, Fan YT (2018). Necroptosis promotes autophagy-dependent upregulation of DAMP and results in immunosurveillance. Autophagy.

[171] Twitty CG, Jensen SM, Hu HM (2011). Tumor-derived autophagosome vaccine: induction of cross-protective immune responses against short-lived proteins through a p62-dependent mechanism. Clin Cancer Res.

[172] Su H, Luo Q, Xie H (2015). Therapeutic antitumor efficacy of tumor-derived autophagosome (DRibble) vaccine on head and neck cancer. Int J Nanomedicine.

[173] Dai Z, Huang J, Lei X (2017). Efficacy of an autophagy-targeted DNA vaccine against avian leukosis virus subgroup J. Vaccine.

[174] Gabai VL, Shifrin VI (2014). Feasibility analysis of p62 (SQSTM1)-encoding DNA vaccine as a novel cancer immunotherapy. Int Rev Immunol.

[175] Noman MZ, Janji B, Berchem G (2012). Hypoxia-induced autophagy: a new player in cancer immunotherapy?. Autophagy.

[176] Teng Y, Ross JL, Cowell JK (2014). The involvement of JAK-STAT3 in cell motility, invasion, and metastasis. Jak-Stat.

[177] Lotze MT, Buchser WJ, Liang X (2012). Blocking the interleukin 2 (IL2)-induced systemic autophagic syndrome promotes profound antitumor effects and limits toxicity. Autophagy.

[178] Li J, Yang D, Wang W (2015). Inhibition of autophagy by 3-MA enhances IL-24-induced apoptosis in human oral squamous cell carcinoma cells. J Exp Clin Cancer Res.

[179] Yang C, Shogren KL, Goyal R (2013). RNA-dependent protein kinase is essential for 2-methoxyestradiol-induced autophagy in osteosarcoma cells. PLoS One.

[180] Pham DC, Chang YC, Lin SR, et al. FAK and S6K1 inhibitor, Neferine, dually induces autophagy and apoptosis in human neuroblastoma cells. Molecules. 2018;23(12). 10.3390/molecules23123110 PMC6321370 30486505

[181] Hahm ER, Sakao K, Singh SV (2014). Honokiol activates reactive oxygen species-mediated cytoprotective autophagy in human prostate cancer cells. Prostate.

[182] Chung SJ, Nagaraju GP, Nagalingam A (2017). ADIPOQ/adiponectin induces cytotoxic autophagy in breast cancer cells through STK11/LKB1-mediated activation of the AMPK-ULK1 axis. Autophagy.

[183] Zhang P, Zheng Z, Ling L (2017). w09, a novel autophagy enhancer, induces autophagy-dependent cell apoptosis via activation of the EGFR-mediated RAS-RAF1-MAP2K-MAPK1/3 pathway. Autophagy.

[184] Sun L, Hu L, Cogdell D (2017). MIR506 induces autophagy-related cell death in pancreatic cancer cells by targeting the STAT3 pathway. Autophagy.

[185] Gong K, Zhang Z, Chen Y (2014). Extracellular signal-regulated kinase, receptor interacting protein, and reactive oxygen species regulate shikonin-induced autophagy in human hepatocellular carcinoma. Eur J Pharmacol.

[186] Fan XJ, Wang Y, Wang L (2016). Salidroside induces apoptosis and autophagy in human colorectal cancer cells through inhibition of PI3K/Akt/mTOR pathway. Oncol Rep.

[187] Li Q, Song XM, Ji YY (2013). The dual mTORC1 and mTORC2 inhibitor AZD8055 inhibits head and neck squamous cell carcinoma cell growth in vivo and in vitro. Biochem Bioph Res Co.

[188] Wang Y, Zhang W, Lv Q (2016). The critical role of quercetin in autophagy and apoptosis in HeLa cells. Tumor Biol.

[189] Lin YC, Lin JF, Wen SI (2017). Chloroquine and hydroxychloroquine inhibit bladder cancer cell growth by targeting basal autophagy and enhancing apoptosis. Kaohsiung J Med Sci.

[190] Simone C (2007). Signal-dependent control of autophagy and cell death in colorectal cancer cell: the role of the p38 pathway. Autophagy.

[191] Zhao X, Fang Y, Yang Y (2015). Elaiophylin, a novel autophagy inhibitor, exerts antitumor activity as a single agent in ovarian cancer cells. Autophagy.

[192] Zhao B, Shen C, Zheng Z (2018). Peiminine inhibits glioblastoma in vitro and in vivo through cell cycle arrest and Autophagic flux blocking. Cell Physiol Biochem.

[193] Papademetrio DL, Lompardia SL, Simunovich T (2016). Inhibition of survival pathways MAPK and NF-kB triggers apoptosis in pancreatic ductal adenocarcinoma cells via suppression of autophagy. Target Oncol.

